# Environmental relevance monitoring and assessment of ochreous precipitates, hydrochemistry and water sources from abandoned coal mine drainage

**DOI:** 10.1007/s10661-024-12858-x

**Published:** 2024-07-04

**Authors:** Tuan Quang Tran, Sylvia Riechelmann, Andre Banning, Stefan Wohnlich

**Affiliations:** 1 Department of Hydrogeochemistry and Hydrogeology, Institute of Geology, Mineralogy and Geophysics, Faculty of Geosciences, Ruhr-University Bochum, Universitätsstraße 150, 44801 Bochum, Germany; 2 Faculty of Geosciences and Geoengineering, Hanoi University of Mining and Geology, No. 18, Pho Vien, Duc Thang Ward, Bac Tu Liem District, Hanoi, Vietnam; 3 Department of Sediment and Isotope Geology, Institute of Geology, Mineralogy and Geophysics, Faculty of Geosciences, Ruhr-University Bochum, Universitätsstraße 150, 44801 Bochum, Germany; 4 Department of Applied Geology, Institute of Geography and Geology, University of Greifswald, Friedrich-Ludwig-Jahn-Str. 17A, 17489 Greifswald, Germany

**Keywords:** Hydrochemistry, Precipitates, Environmental monitoring, Oxygen and hydrogen stable isotopes, Mine water, Ruhr coalfield

## Abstract

This study investigated the mineralogical and chemical characteristics of ochreous precipitates and mine water samples from abandoned Upper Carboniferous hard coal mines in an extensive former mining area in western Germany. Mine water characteristics have been monitored and assessed using a multi-methodological approach. Thirteen mine water discharge locations were sampled for hydrochemical analysis, with a total of 46 water samples seasonally collected in the whole study area for stable isotopic analyses. Mineralogical composition of 13 ochreous precipitates was identified by a combination of powder X-ray diffraction (XRD), Fourier-transform infrared spectroscopy (FTIR), and field emission scanning electron microscopy (FE-SEM/EDS). Results showed that abandoned mine drainage was characterized by circumneutral pH, Eh values ranging from 163 to 269 mV, relatively low concentrations of Fe and Mn, and was dominated by HCO_3_^−^  > SO_4_^2−^  > Cl^−^  > NO_3_^−^ and Na^+^  > Ca^2+^  > Mg^2+^  > K^+^. Goethite and ferrihydrite were the dominant precipitated Fe minerals, with traces of quartz, dolomite, and clay minerals. Some metal and metalloid elements (Mn, Al, Si, and Ti) were found in the ochreous sediments. The role of bacteria in the formation of secondary minerals was assessed with the detection of *Leptothrix ochracea*. The δ^18^O and δ^2^H values of mine water plotted on and close to the GMWL and LMWLs indicated local derivation from meteoric water and represented the annual mean precipitation isotopic composition. Results might help to develop strategies for the management of water resources, contaminated mine water, and public health.

**Supplementary Information:**

The online version contains supplementary material available at 10.1007/s10661-024-12858-x.

## Introduction

Mine drainage is one of the numerous issues occurring at active and decommissioned mine sites. Evaluating the effects of historical mining activities on the surface is a matter of relevance. Mine drainage may exist for decades after mining, leading to major long-term challenges (Gagliano et al., 2004). It can affect local groundwater and surface water quality (Bowell & Bruce, 1995). After active mining ceases, shafts, adits, waste material, and tailings are left behind. Ochreous precipitates from mine water deposit on flow beds in the drainage adits and contain a diverse group of iron-oxyhydroxide minerals. Oxidation of disulfides (e.g., pyrite that is found in coal mines) and Fe(III) hydroxy-sulfate minerals have been identified as one of the most important inorganic pollution sources for groundwater and surface waters (Azzali et al., 2014; Bao et al., 2018).

In mine water, the formation of ochreous precipitates varies depending on different conditions such as pH, redox reactions, desorption, absorption, and ion exchange reactions (Kumpulainen et al., 2007; Michalková & Šubrt, 2015). Singer and Stumm (1970), and Stumm and Morgan (1996) explained the processes of disulfide oxidation, including the production of acidic mine drainage and the formation of precipitates. The acidity of mine water is reduced when mine water interacts with buffer minerals (e.g., carbonates and silicates) (Nordstrom, 2011; Roisenberg et al., 2016). The precipitation of iron oxyhydroxides occurs when the mine water pH increases from a low value (e.g., pH < 5) to a higher value (pH 5–8) (Bowell & Bruce, 1995). Simultaneously, when the dilution or neutralization is sufficient, it affects the formation of secondary minerals from the Fe-rich sulfate solution. The ochreous compounds are mainly made up of Fe compounds that range in color from yellow to reddish-brown (Equeenuddin et al., 2010).

Many studies indicate that minerals of ochreous precipitates in coal mine drainage show variations from amorphous to well-crystalline forms. There are different precipitates of Fe(III) minerals such as goethite (α-FeOOH), ferrihydrite (Fe_2_O_3_.9H_2_O), schwertmannite (Fe_8_O_8_(OH)_6_SO_4_), hematite (α-Fe_2_O_3_), and jarosite (KFe_3_(SO_4_)_2_(OH)_6_), which form at different field pH conditions and SO_4_^2−^ concentrations of mine water (Kumpulainen et al., 2007; Peretyazhko et al., 2009). These minerals are a product of the iron oxidation process, which is catalyzed by the activity of microorganisms (Kim et al., 2003). Jarosite forms in an acidic environment (pH < 3) with a high SO_4_^2−^ concentration, and schwertmannite also commonly precipitates under acidic conditions (pH 3–4). Goethite and ferrihydrite usually form in many different environments, from slightly acidic to circumneutral pH and alkaline conditions (Dold, 2003; Gao & Schulze, 2010; Schwertmann & Carlson, 2005). Goethite represents a stable iron phase in precipitates and is easily identifiable by X-ray diffraction analysis (Parafiniuk & Siuda, 2006). In many cases, Fe(III) precipitates from mine water are also mixtures of different minerals because of the transformation processes to thermodynamically more stable phases (Michalková & Šubrt, 2015). This transformation occurs with time into more stable minerals. For example, schwertmannite is formed in low pH conditions and then transformed into goethite when the pH of the mine water increases (Gagliano et al., 2004; Acero et al., 2006). However, the ferrihydrite formation will be more dominant if the organic carbon content is high in the mine water (Kumpulainen et al., 2007; Michalková & Šubrt, 2015). Ferric oxyhydroxides can precipitate in the mine adits or the mine drainage channels (Máša et al., 2012).

Secondary minerals can play an important role in controlling the mobility and transport of contaminants. In circumneutral pH, precipitation of Fe secondary minerals leads to co-precipitation of other metals (Abongwa et al., 2020; Bowell & Bruce, 1995). When the pH of the solution increases, other aqueous metal species also tend to precipitate, leading to the adsorption of contaminants from mine water (Karapınar, 2016). Goethite and ferrihydrite may have a role in the removal of trace elements from the solution (Máša et al., 2012; Peretyazhko et al., 2009) and incorporate trace elements into the Fe hydroxide structure (Acero et al., 2006; Kumpulainen et al., 2007). As a result, it restricts the release of dissolved metals from mine water runoff (Bowell & Bruce, 1995; Kumpulainen et al., 2007). Winland et al. (1991) proved that concentrations of metal elements are controlled by adsorption and co-precipitation in coal mine water solutions in Ohio, USA. Moreover, microbial activity in mine drainage has a crucial role in the formation of secondary minerals and mineralogical transformation with available organic carbon (Bao et al., 2018; Equeenuddin et al., 2010).

Oxygen and hydrogen stable isotopic compositions provide important information regarding the recharge sources of groundwater. The isotopic signatures in rainwater may be compared to the stable isotopic compositions of groundwater to track down the recharge source and evaluate recharge mechanisms (Clark & Fritz, 1997). In the case that the isotopic compositions of groundwater plot close to the meteoric water line, meteoric water is identified as a sure source of groundwater (Vermeulen et al., 2014). Moreover, isotopic techniques have been widely approached for some decades in studying mine water characteristics. In particular, many researchers used environmental isotopes to identify water sources in abandoned coal mines in China and the USA (Gammons et al., 2010; Qu et al., 2018). Thus, gaining more detailed knowledge about the mine water source is essential to managing and preventing pollution.

In the study area, the ochreous precipitates from mine drainage were geochemically characterized as Fe precipitates that were noticed in previous studies, but they are rarely published. This represents an important gap currently in knowledge given the potential role of Fe precipitates in metal budgets and contaminant mobility. Understanding the recharge source, transport, and precipitates are worthy studied objects for environmental and post-mining management and remediation. Furthermore, the mine drainage from abandoned coal mines might also be a water source for use in the region. Therefore, this study was developed with three main goals: (1) to assess mine water hydrochemical characteristics at discharge locations where ochreous precipitation formed; (2) to identify the mineral composition of ochreous precipitates formed in various abandoned coal mines; and (3) to help determine the recharge source of mine water in the abandoned coal mines using oxygen and hydrogen stable isotopes. This study will help water resource managers better understand the mine water composition after the completion of coal exploitation and plan to exploit and use this water resource. Considering the global trend to move away from coal mining in the course of the energy transition, the present study is of far more than regional relevance; its results and implications are relevant to numerous areas worldwide encountering present and future post-mining water quality issues.

## Study area

**Fig. 1:**
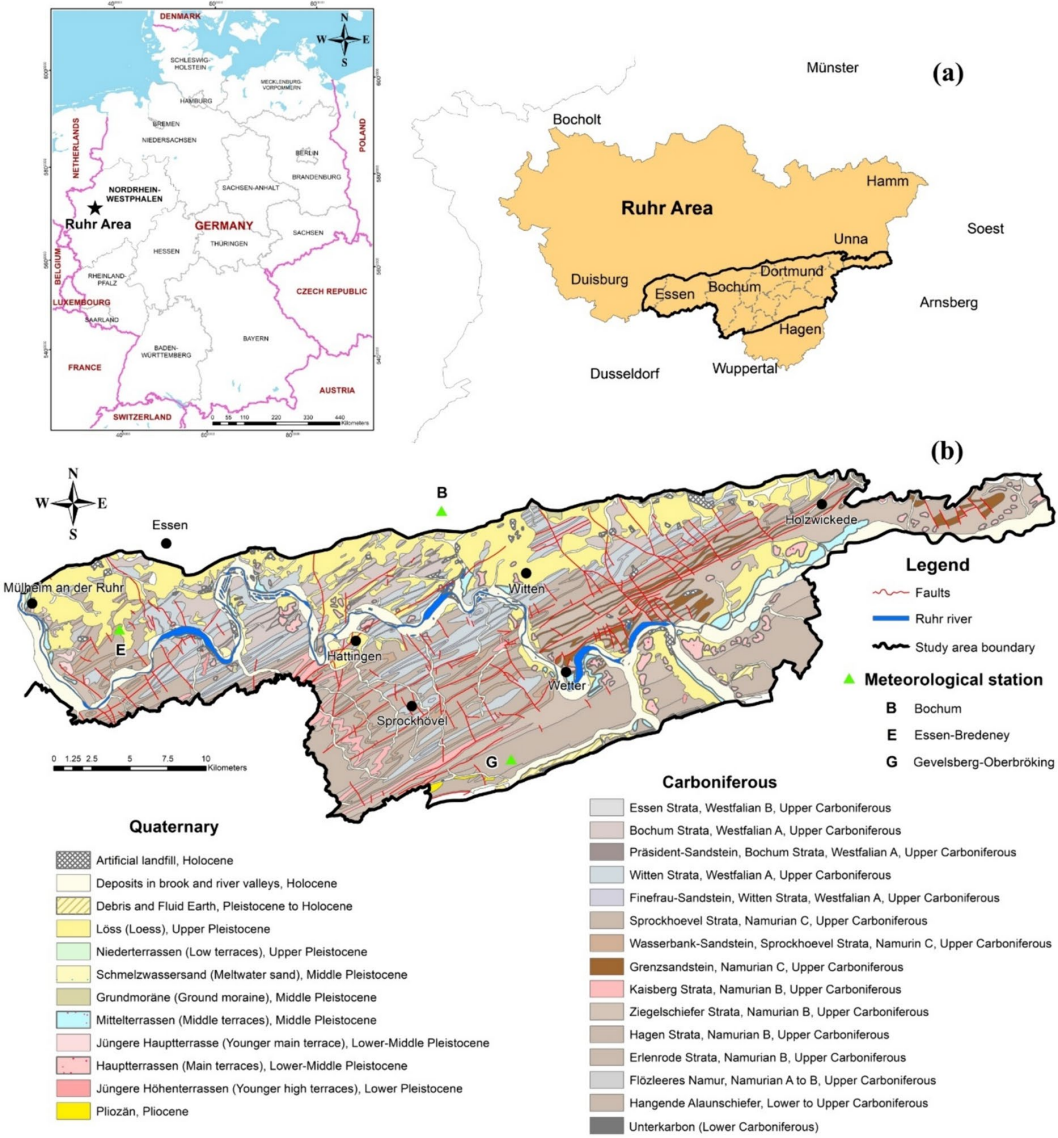
**(a)** Location map and (**b**) geological map of the study area (modified from GD.NRW-WMS, 2017)

The study area is located in a region of abandoned coal mines where active water supply plants exist around the lower Ruhr River area in Germany, occupying an area of approximately 754 km^2^ (Fig. 1). This area has a long history of coal mining, stretching back to the thirteenth century. Abandoned coal mines resulted from early shallow mining, which mostly took place in the nineteenth century. In the 1960s, all hard coal mining activities were decommissioned in the study area (Drobniewski et al., 2017) when active mining moved further north. The river Ruhr flows from east to west through the study area and is a major surface water source in the area. In general, the elevation ranges from about 20 to 330 m above sea level (a.s.l.) and is characterized by shallow hills. The highest elevations are located in the south (in the towns Hattingen and Sprockhövel), whereas the lowest elevation is located in the west of the area (in Mülheim an der Ruhr) (Fig. 1). The climate is characterized by fully humid conditions with mild winters and warm summers (Cfb climate in the Köppen classification; LANUV, 2018; Peel et al., 2007). According to data from the meteorological stations Bochum, Essen-Bredeney, and Gevelsberg-Oberbröking (2008–2018), the annual average air temperature is around 10.4 °C and the annual average rainfall varies from 849.0 to 954.4 mm (DWD, 2019).

In terms of geology, the Upper Carboniferous strata crop out directly on the surface in the study area (Fig. 1b). In the coal-bearing Carboniferous formations, coal seams started in the Namurian C strata and reached their maximal capacity in the Westphalian A and B (Drozdzewski, 1993; Littke & Haven, 1989). The Carboniferous strata are characterized by a cyclothem structure and comprise alternate layers of sandstone, mudstone, siltstone, and claystone layers that contain feldspar (albite, anorthite), clay minerals (kaolinite, chlorite, biotite), siderite, dolomite, calcite, quartz, and pyrite (Strehlau, 1990; Wisotzky, 2018) with interspersed coal seams (Coldewey & Semrau, 1994). The Erlenrode, Hagen, Kaisberg, and Ziegelschiefer strata (Namurian B deposits) crop out in the western, southern, and eastern parts of the area (Fig. 1), including siltstone, claystone, mudstone, sandstone layers, subordinate sandstone, and quartz, with mica-rich minerals and local conglomerates (GD.NRW-WMS, 2017). The Sprockhövel stratum (Namurian C deposits) mostly covers parts in Hattingen, Sprockhövel, Wetter, Witten, Dortmund, Essen, and Fröndenberg/Ruhr, accounting for 30–32% of the exposed parts (Fig. 1). This formation comprises siltstone, dark gray sandstone (feldspar, quartz, calcite, pyrite), and mudstone layers, with hard coal seams (GD.NRW-WMS, 2017). The Bochum and Witten strata (Westphalian A deposits) contribute to the Sprockhövel and Holzwickede areas, the central part of Dortmund, and the south of Essen and Bochum. The Witten stratum is exposed and concentrated mainly in the central part of the study area, accounting for about 15–18% of the outcrops. It is composed of sandstone, subordinate sandstone layers (from medium to coarse-grained), thick-banked, gray conglomerate layers that are interspersed, and dark gray siltstone and mudstone layers (from weak to very sandy content), with numerous hard coal seams. The Bochum stratum is mainly formed by dark gray siltstone (with the sandy content changing at different rates), with silica, calcite, and iron oxides being the most common cementing minerals for siltstone. Furthermore, the Bochum stratum consists of mudstone (from weak to very sandy content) interspersed by light gray conglomerate layers, which are accompanied by sandstone and subordinate sandstone layers (from medium to coarse-grained). Feldspars and micas are common minerals in the host rocks, and numerous coal seams are deposited in this formation. The Essen stratum (Westphalian B deposit) is widely spread in the study area; however, it has limited exposure. It consists of siltstone and mudstone layers with numerous hard coal seams (GD.NRW-WMS, 2017). Quaternary deposits (Pliocene and Pleistocene) with a thin thickness are contributed by a small part, overlying the Upper Carboniferous formations, including loess clay to coarse-sandy terrace gravel and boulder clay to loess (Coldewey & Semrau, 1994). Holocene deposits (clayey, fine sand, silt with deposits of melt-water and loess, and artificial landfill materials) are distributed along the Ruhr River and its tributaries (Fig. 1; GD.NRW-WMS, 2017).

In the study area, the porous aquifer in Quaternary deposits along the Ruhr River valley is one of the most important aquifers, as groundwater in this aquifer is used as the main source of water supply in the metropolitan area. The aquifer is characterized by loess, silt, sand, and gravel deposits with a thickness ranging from 8 to 10 m (Wisotzky & Wohnlich, 2010). The hydraulic conductivity varies from 1 × 10^−5^ to 1 × 10^−2^ m s^−1^, indicating moderate to high permeability. The fissured and fractured Upper Carboniferous aquifer is spread over the whole study area, and most of the Carboniferous layers act as an aquitard. The hydraulic conductivity changes between 1 × 10^−4^ and 1 × 10^−9^ m s^−1^, indicating moderate to very low permeability (GD.NRW-WMS, 2017).

## Data collection and methodology

### Field description and sampling

**Fig. 2:**
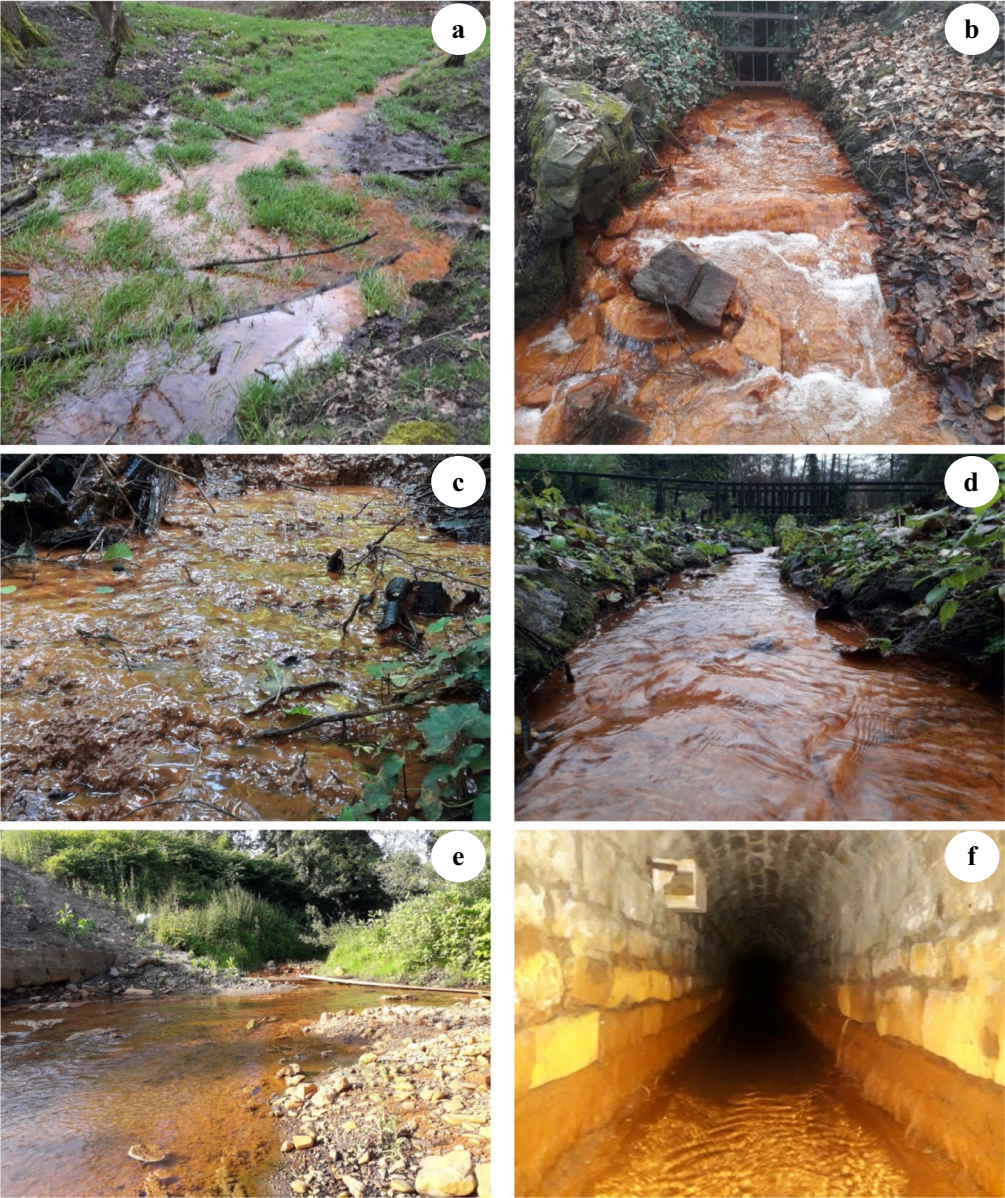
Selection of ochreous precipitates from mine water in the study area corresponding to the approximate width of the streams/discharges: at the adits (**a**) Rösche von Mit Gott gewagt (4–5 m), (**b**) Pauline Stollen (1.0–1.5 m), (**c**) ferric hydroxide terraces in Quelle Schacht 1 entrance (1.0–1.2 m); (**d**) Roter Bach (0.7–1.0 m), (**e**) at the entrance of Franziska Erbstollen (4–6 m), and (**f**) St. Johannes Erbstollen (1.0–1.2 m) (cf. Figure 3 for locations)

**Fig. 3:**
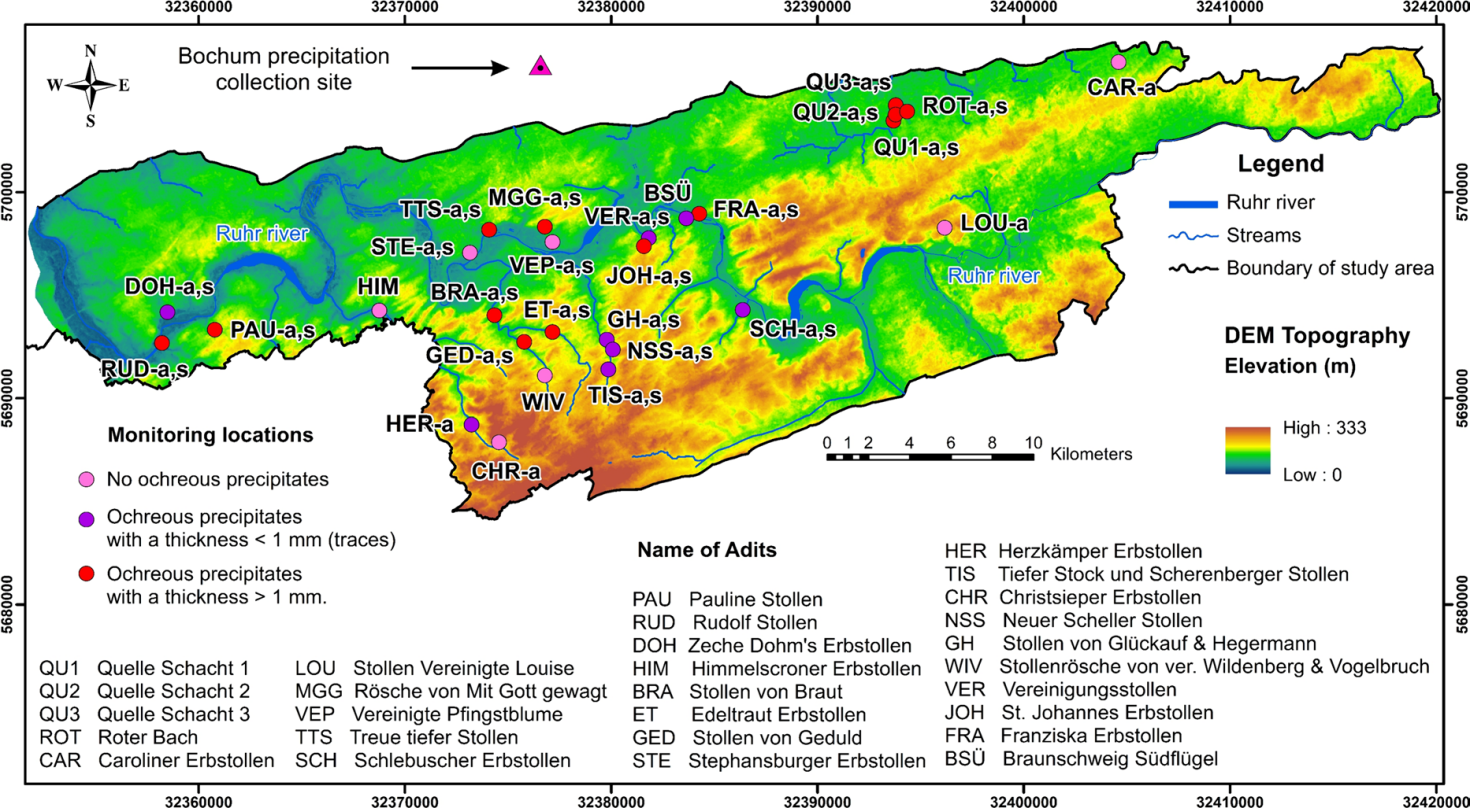
Sampling locations on a DEM topographic map (UTM Zone N32); Red circles illustrate the same sites of 13 water chemistry and 13 precipitate samples; 25 and 21 stable isotope water samples were collected in two seasons at sites with the symbols ‘a’ for autumn and ‘s’ for summer, respectively; A rainwater collection site (triangle) in Bochum is marked

Many abandoned mines have been left in the coal-bearing Upper Carboniferous formations with historical water drainage (adits) still existing. Some sites are difficult to access or have no water, whereas there are roughly 28 discharge locations where mine water discharges to the surface in different seasons (Figs. 2 and 3). Thirteen of these adits show secondary iron mineral precipitates with a thickness of > 1 mm, including adits Rudolf Stollen (RUD) and Pauline Stollen (PAU) in Essen, Rösche von Mit Gott gewagt (MGG) and Treue tiefer Stollen (TTS) in Bochum, Quelle Schacht 1 (QU1), Quelle Schacht 2 (QU2), Quelle Schacht 3 (QU3) and Roter Bach (ROT) in Dortmund, Franziska Erbstollen (FRA) and St. Johannes Erbstollen (JOH) in Witten, and adits Edeltraut Erbstollen (ET), Stollen von Geduld (GED), and Stollen von Braut (BRA) in Sprockhövel. Visible iron oxides and hydroxide precipitates were identified at the adit entrances. Simultaneously, these precipitates deposit and coat the surface of the bottom of the rivulets with a variable thickness (Fig. 2). At these locations, the maximum thickness of precipitates can be observed at the Franziska Erbstollen’s entrance with a precipitate bed of about 80 cm in-depth and covering a 4–6 m width. Otherwise, the precipitates are not ubiquitous at the remaining seven and eight sites, with the thickness of precipitates < 1 mm (traces) and no precipitates (Fig. 3). In the area, the main settings for secondary mineral formations are unconsolidated precipitates, flocs, and loose suspensions forming from mine water solutions. Consolidated precipitates (hard layered crusts of precipitates) can be observed at the entrance of Franziska Erbstollen.

Water samples for chemical analyses were collected from 13 drainage adits of abandoned coal mines in the study area showing ochreous precipitates (thickness of the precipitates > 1 mm) (Figs. 2 and 3). Water samples were collected at the same sites as ochreous precipitate samples. All precipitates were taken at the bottom of streams during the period of lowest drainage stream flows (in autumn). Forty-six mine water samples were taken for δ^18^O and δ^2^H isotopic analyses, while 13 rainwater samples were collected monthly from March 2020 to March 2021 in Bochum (94 m a.s.l., Fig. 3) for these analyses. Most of the mine water samples were collected in autumn 2019 (25 samples), and most of these sites were revisited for a second sampling in summer 2020 (21 samples) to identify the origin of mine water and assess the change in isotopic composition seasonally. Due to the lack of flow conditions and inaccessibility, the number of obtained samples was different.

### Water sample collection

The field parameters pH, water temperature, and electrical conductivity (EC) were measured using a Multi 350i meter, equipped with a SenTix 41 electrode for pH and a TetraCon 325 electrode for temperature and EC values, respectively. The oxidation–reduction potential (ORP) was determined using a pH 340 with a SenTix PtR electrode and Eh values were converted to the standard hydrogen electrode (SHE) (Wolkersdorfer, 2008). Dissolved oxygen (O_2_) was measured using an Oxi 197 with a CellOx 325 sensor (all WTW, Weilheim, Germany). Mine water samples were collected in 250 mL high-density glass bottles previously washed with distilled water (3 times) for HCO_3_^−^ analysis. In the field, water samples for major ions, dissolved metal (Fe, Mn, Cd, Cu, and Pb), and metalloid (As) analyses were filtered through 0.45 μm Millipore cellulose acetate membrane filters into 50 mL PE bottles, filled to the top, and capped tightly. To avoid Fe hydroxide precipitation, samples for cation and minor metals analyses were acidified to pH < 2 using ultra-purified HNO_3_ (≥ 65%). Samples were brought back to the laboratory and maintained in a fridge at 4 °C until analysis. Rainwater samples were collected using a rainfall collector (a rain bucket). All rainwater and mine water samples for δ^18^O and δ^2^H isotopic analyses were filtered in the field through sterile 0.2 μm membrane filters by a syringe and placed into 2 mL glass vials without headspace, closed with screw caps and a septum. To avoid evaporation, parafilm paper was used to wrap the bottle caps of the sample bottles. Afterward, these water samples were stored in a refrigerator at 5 °C in the laboratory until analysis.

### Ochreous precipitate samples and preparation

Precipitate samples were collected using a plastic spoon or sampling shovel, and gloves, and deposited in sealed plastic bags or glasses, and they were stored in a dark box to minimize atmospheric contamination. Afterward, samples were transferred to the laboratory to prevent mineralogical changes. Before drying, coarse organic material, such as roots and fallen leaves, was removed. Samples were controlled-dried at 40 °C in an oven for further study. After drying, samples were ground with an agate mortar and pestle and manually sieved to remove the large detrital materials in the laboratory using the 63 μm and 0.1 mm sieves for FTIR and XRD analyses. Samples were dried and only crushed for FE-SEM/EDS analysis.

### Analytical techniques and data collection

All samples were analyzed in different laboratories at the Institute of Geology, Mineralogy and Geophysics, Faculty of Geosciences, Ruhr-University Bochum.

#### Water chemical and stable isotopic analyses

The HCO_3_^−^ concentration was measured twice to triplicate for each sample in the laboratory using the HCl acid titration method. Major anions (SO_4_^2−^, Cl^−^, and NO_3_^−^) were analyzed using an ion chromatography system, model ICS-1000 (DIONEX-Thermo Fisher Scientific GmbH, Dreieich, Germany). Major cations (Ca^2+^, Mg^2+^, Na^+^, and K^+^) were analyzed using a CD 25 conductivity detector system (DX 500, column CS12A; DIONEX-Thermo Fisher Scientific GmbH, Dreieich, Germany). Analytical accuracy was estimated via an electrical charge balance (< 5%). The dissolved metals and metalloid in water samples were determined using inductively coupled plasma optical emission spectroscopy (ICP-OES, Optima 8300 DV, Perkin Elmer), with 0.1 mg L^−1^ for Fe and Mn, 10 μg L^−1^ for Cd, Cu, Pb, and 20 μg L^−1^ for As, respectively, in the lower limit concentrations of these element detections. Water samples for oxygen and hydrogen stable isotopic analyses were placed in an autosampler AS 3000 (Thermo Fisher Scientific) and isotopic ratios were determined using a CF-IRMS (Continuous-flow-isotope-ratio-mass-spectrometer) 253 plus (Thermo Fisher Scientific) equipped with a ConFloIV and a TC/EA (High-Temperature Conversion Elemental Analyzer; both Thermo Fisher Scientific) by converting H_2_O into H_2_ and CO gas in the reactor filled with glassy carbon granulate. The TC/EA Reactor temperature was set to 1400 °C, while the TC/EA GC-Column temperature was 90 °C. The autosampler syringe size was 10 µL. The syringe was rinsed once before the sample injection and once after injection with deionized water. It was rinsed three times with the sample before a 1.5 µL sample volume was injected into the reactor. Furthermore, 3 plunger strokes were performed before sample uptake for injection. The pre-injection dwell time was 2 s, while the post-injection dwell time was 1 s. Standardization of two in-house reference solutions was accomplished using VSMOW2, SLAP2, and GRESP reference solutions (IAEA). The results are reported relative to the VSMOW standard (Vienna Standard Mean Ocean Water) in permil (‰) and the analytical precisions (± 1 σ) were ± 0.17‰ for δ^18^O and ± 1.1‰ for δ^2^H, respectively.

#### Mineralogical analysis

Mineralogical composition identification of precipitate samples was carried out using an EMPYREAN Diffractometer system. Copper Kα radiation and a PIXcel1D detector equipped with a vertical goniometer were used for the XRD at 45 kV and 40 mA. To decrease the interferences of any fluorescence, the detector was specifically adjusted by limiting the energy window to exclude most of the diffuse scattering. Powder samples with randomly orientated crystals were scanned from 4 to 80° 2*θ* with 0.01° steps and a counting rate of 2 s/step because the samples are poorly crystalline. To identify the peaks of different mineral phases, this study used MATCH! 3 software (version 3.11.4.199) and the COD database (Crystal Impact, 2021). The quantification of the phases was performed using the FullProf software package for Rietveld refinement. The FTIR analysis was conducted using a Fourier-transform infrared (FTIR) spectrometer (Nicolet 6700) equipped with a DLaTGS detector (Thermo Scientific). Samples were measured in a humidity-free atmosphere at room temperature. The FTIR spectra were obtained in the wavenumber range of 400–4000 cm^−1^ with a spectral resolution of 2 cm^−1^ to analyze the functional groups present on the surface. All precipitate samples were also examined for their mineralogical composition using field emission scanning electron microscopy (FE-SEM) to identify accessory minerals and particle morphology. This system operated with an acceleration voltage of 0.02 to 30 kV. Samples were coated with a thin gold film to improve conductivity. Energy-dispersive spectrometry (EDS) was used to identify the major and minor elements present in the samples. FE-SEM iteratively gives qualitative chemical analysis results to identify the minerals present. The color of the precipitate samples was described by comparing dried and homogeneously ground samples using Munsell soil color charts (Munsell, 2009). The geochemical modeling of mine water predicted the saturation states of the observed precipitate mineral phases using PHREEQC geochemical software (version 3.6.2) (Parkhurst & Appelo, 2013) with the WATEQ4F database. The aqueous species of dissolved elements and mineral saturation indices (SI) for mine water samples with respect to relevant solid phases were calculated. The solubility of ferrihydrite from Yu et al. (1999) was added to the thermodynamic database of PHREEQC for reference.

## Results

### Mine water hydrochemistry

The statistical results of the mine water hydrochemical analysis of 13 collected samples are summarized in Table 1. The field pH values did not change considerably between water samples, showing a circumneutral condition. Water temperature and EC values were variable, and the measured EC values at some adits located in the northeastern part of the study area, including Quelle Schacht 1, Quelle Schacht 2, Quelle Schacht 3, Roter Bach, and Franziska Erbstollen, were higher than those of the remaining adits. Comparing different monitoring locations, the most dominant anion was HCO_3_^−^, followed by SO_4_^2−^, and their average concentrations were characterized by HCO_3_^−^  > SO_4_^2−^  > Cl^−^  > NO_3_^−^. Regarding the cationic composition, Na^+^ and Ca^2+^ were dominant cations, and mean cation concentrations were determined to be Na^+^  > Ca^2+^  > Mg^2+^  > K^+^ in water samples. The SO_4_^2−^, HCO_3_^−^, Na^+^, and Ca^2+^ concentrations at Quelle Schacht 1, Quelle Schacht 2, Quelle Schacht 3, and Roter Bach were relatively higher than those of other locations. Water samples with higher Fe and Mn concentrations were found in Dortmund (Quelle Schacht 1, Quelle Schacht 2, and Quelle Schacht 3), Bochum (Rösche von Mit Gott gewagt), Hattingen (Stollen von Geduld), and Witten (Franziska Erbstollen and St. Johannes Erbstollen). The Fe and Mn concentrations of Stollen von Braut were the lowest (Table 1).

**Table 1:** Field measurement data and hydrochemical analyses of mine water from 13 selected adits in the study area (in autumn 2019)

Sample ID	Field measurement data						Chemical compositions, mg L^−1^									
	pH, –	T, °C		Eh, mV	EC, μS cm^−1^	O_2_, mg L^−1^	SO_4_^2−^	HCO_3_^−^	Cl^−^	NO_3_^−^	Ca^2+^	Mg^2+^	Na^+^	K^+^	Fe	Mn
RUD	6.8	11.1		207	536	0.73	52.1	265.3	16.0	< 0.1	60.2	24.9	12.4	8.2	2.6	0.6
TTS	6.6	11.2		–	–	–	55.4	253.6	37.0	< 0.1	65.5	28.3	23.8	8.2	1.6	0.5
BRA	7.0	12.3		269	694	2.79	85.3	325.3	25.0	0.5	59.9	31.7	42.3	10.0	0.5	0.4
JOH	6.7	11.7		231	747	7.89	174	253.2	12.0	0.9	86.9	41.1	14.7	7.9	2.9	0.85
PAU	6.7	11.3		245	757	1.42	134	314.1	28.0	1.9	84.0	39.4	22.0	9.5	2.1	0.9
MGG	6.7	11.7		209	808	0.83	76.5	352.1	46.0	< 0.1	88.2	37.5	17.0	6.4	5.2	1.1
ET	6.7	14.4		232	831	1.85	147	337.5	18.0	0.6	54.6	29.2	80.4	10.9	2.3	0.8
GED	7.0	14.1		177	861	3.33	103	426.3	17.0	0.5	50.5	27.0	105	11.9	4.2	0.4
FRA	6.9	15.7		185	1415	2.62	185	607.0	67.1	< 0.1	88.9	35.2	186	12.7	2.8	0.5
QU3	6.9	12.5		195	1731	0.09	341	631.4	59.2	< 0.1	112	45.9	245	10.5	4.6	1.0
QU1	6.8	12.7		214	1799	1.03	335	649.7	60.7	< 0.1	107	44.2	237	10.5	3.2	0.6
QU2	6.8	12.9		167	1737	0.12	337	631.5	59.4	< 0.1	108	44.2	268	10.7	3.0	0.6
ROT	6.8	14.7		163	1762	0.12	344	652.0	61.3	< 0.1	93.9	40.3	257	9.9	2.6	0.6
Average	6.8	12.8		208	1140	1.90	182	438.4	39.0	0.9	81.5	36.1	116	9.8	2.9	0.7
SD	0.1	1.5		32.5	500	2.18	116.3	167.7	20.7	0.6	21.3	7.2	105.7	1.7	1.2	0.2

*SD* standard deviation; (–): not determined. The detection limit for major ions is < 0.1 mg L^−1^ and for Fe and Mn, this limit is < 0.01 mg L^−1^; Cd, Cu, and Pb concentrations < 10 μg L^−1^; As concentration < 20 μg L^−1^

**Fig. 4:**
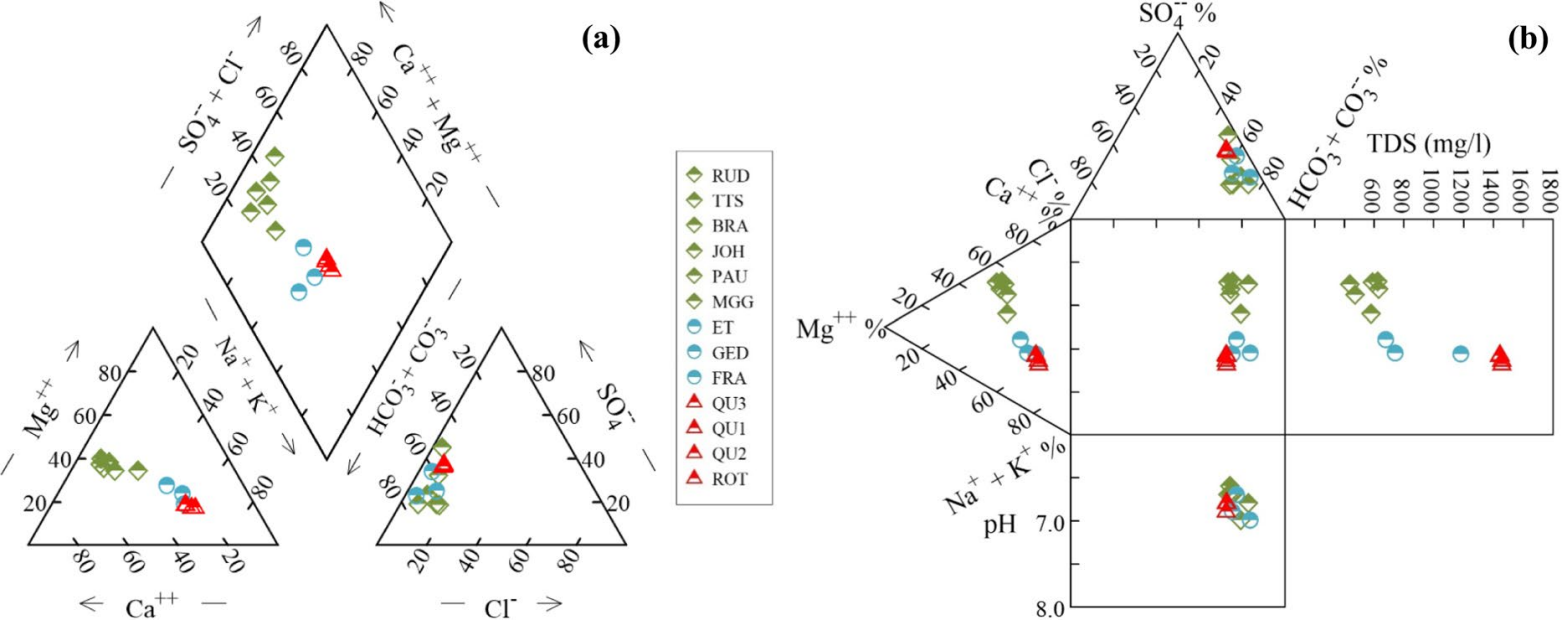
(**a**) Piper diagram and (**b**) Durov diagram of 13 mine water hydrogeochemical data

The differences in major ion concentrations of water samples are plotted on Piper and Durov diagrams (Fig. 4). Water samples are classified into three distinguished groups (3 different colors in the figure) based on total dissolved solids (TDS) and similarity in major ion composition. The first group is distributed in the western part of the study area, including six samples such as RUD, PAU, BRA, TTS, MGG, and JOH, which accounted for 46.2% of the total water samples collected. In general, this group is mainly defined by the lowest values of major ion concentrations (except for Ca^2+^ and Mg^2+^ concentrations that are higher than those in group 2), temperature, EC, and TDS, but NO_3_^−^ and O_2_ concentrations are the highest. The second group is formed by three samples (GED, ET, and FRA) and is located in the central section of the study area, accounting for 23.1% of samples. Compared to the first group, HCO_3_^−^, SO_4_^2−^, Cl^−^, Na^+^, and K^+^ concentrations in the second group are higher than those in the first group, while NO_3_^−^, Ca^2+^, and Mg^2+^ concentrations are lower. Finally, the third group is represented by four samples (QU1, QU2, QU3, and ROT) and accounts for 30.8% of the samples. The samples of group 3 with HCO_3_^−^ and Na^+^ concentrations are observed in the highest proportion, followed by those of concentrations in groups 1 and 2. Most ion concentrations are the highest, while the NO_3_^−^ concentration is absent in this group.

**Fig. 5:**
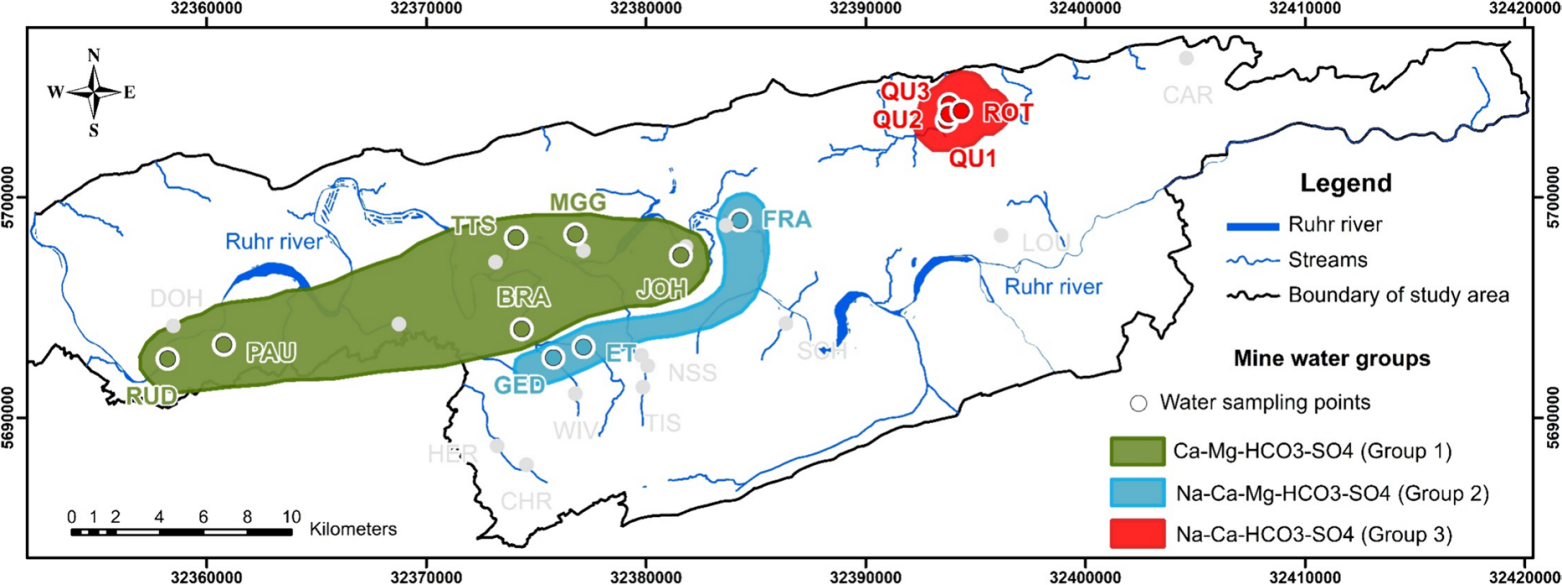
The spatial distribution of three mine water groups from water samples

Based on the mean TDS values of groups, the spatial distribution relating to three different water types is recognized from the ochreous precipitated locations of abandoned coal mines in the study area (Fig. 5). The water type of group 1 is the Ca-Mg-HCO_3_-SO_4_ type, with an average TDS of 562 mg L^−1^, revealing that mine water is freshwater. Group 2 has an average TDS value of 871 mg L^−1^ (freshwater) and presents the Na-Ca-Mg-HCO_3_-SO_4_ type. Samples in group 3 are characterized by the highest TDS, with a mean TDS value of 1456 mg L^−1^ (brackish water) (Freeze & Cherry, 1979), showing a water type of Na-Ca-HCO_3_-SO_4_. In general, waters whose composition is characteristic of shallow-circulation groundwater have a relatively low TDS (green points), while mine water has an elevated TDS (red points). Mine water, whose ratio of major constituents is indicative of deeper circulation groundwater and transitional water (group 2, blue points). The water types change gradually from Ca-Mg-HCO_3_-SO_4_ type (group 1) to Na-Ca-Mg-HCO_3_-SO_4_ type (group 2) and Na-Ca-HCO_3_-SO_4_ type (group 3).

According to the saturation state of mineral phases, the aqueous species and SI values for each water sample were computed by PHREEQC geochemical modeling. The ionic balances are within the acceptable error range of ± 5% (Table S1; AWWA, 1998; Hem, 1985). Ionic strength calculated values for mine waters range from 0.009 to 0.026 mol/kgw, with an average of 0.016 mol/kgw. Sulfate complexes of Fe(III) are minor and present as FeSO_4_^+^, Fe(SO_4_)_2_^−^, and FeHSO_4_^2+^. Under circumneutral pH in the study area, hydroxide species such as Fe(OH)^2+^ and Fe(OH)^4−^ are dominant. Mn is present either as free aqueous ions or as sulfate and bicarbonate species. Saturation indices show that all water samples are commonly saturated and oversaturated with respect to most Fe phases (Table S1). The Fe minerals (e.g., goethite and ferrihydrite) were possible to be precipitated from mine drainage in the study area. The amorphous ferric hydroxide Fe(OH)_3_(a) was saturated with SI values ranging from 0.27 to 2.10, while the SI values of Fe_3_(OH)_8_ ranged from − 1.13 to 2.33. The SI values of low crystalline minerals were greater than zero, for example, ferrihydrite Fe_2_O_3_.9H_2_O (SI: 6.01 to 9.68), and goethite FeOOH (SI: 5.78 to 7.52). Seven water samples document SI values of siderite exceeding + 0.2, implying these samples are supersaturated with respect to this mineral (Merkel & Planer-Friedrich, 2005). Jarosites were undersaturated with a different variation in saturation indices in the water samples (Table S1). For Mn phases, PHREEQC calculations indicate that some samples were saturated or in equilibrium (SI ≈ 0) with respect to rhodochrosite (MnCO_3_) (e.g., Quelle Schacht 1, Quelle Schacht 2, Quelle Schacht 3, Roter Bach, and Rösche von Mit Gott gewagt samples). Otherwise, the most determined SI values of Mn phases were below − 0.2, water samples were undersaturated in relation to the corresponding Mn minerals (Merkel & Planer-Friedrich, 2005). The SI of calcite, dolomite, and aragonite in several samples fluctuated within ± 0.5 intervals. Conversely, all samples were undersaturated concerning anhydrite and gypsum, with SI values < 0 in the modeling calculation (Table S1).

### Precipitate characteristics

#### XRD results

**Fig. 6:**
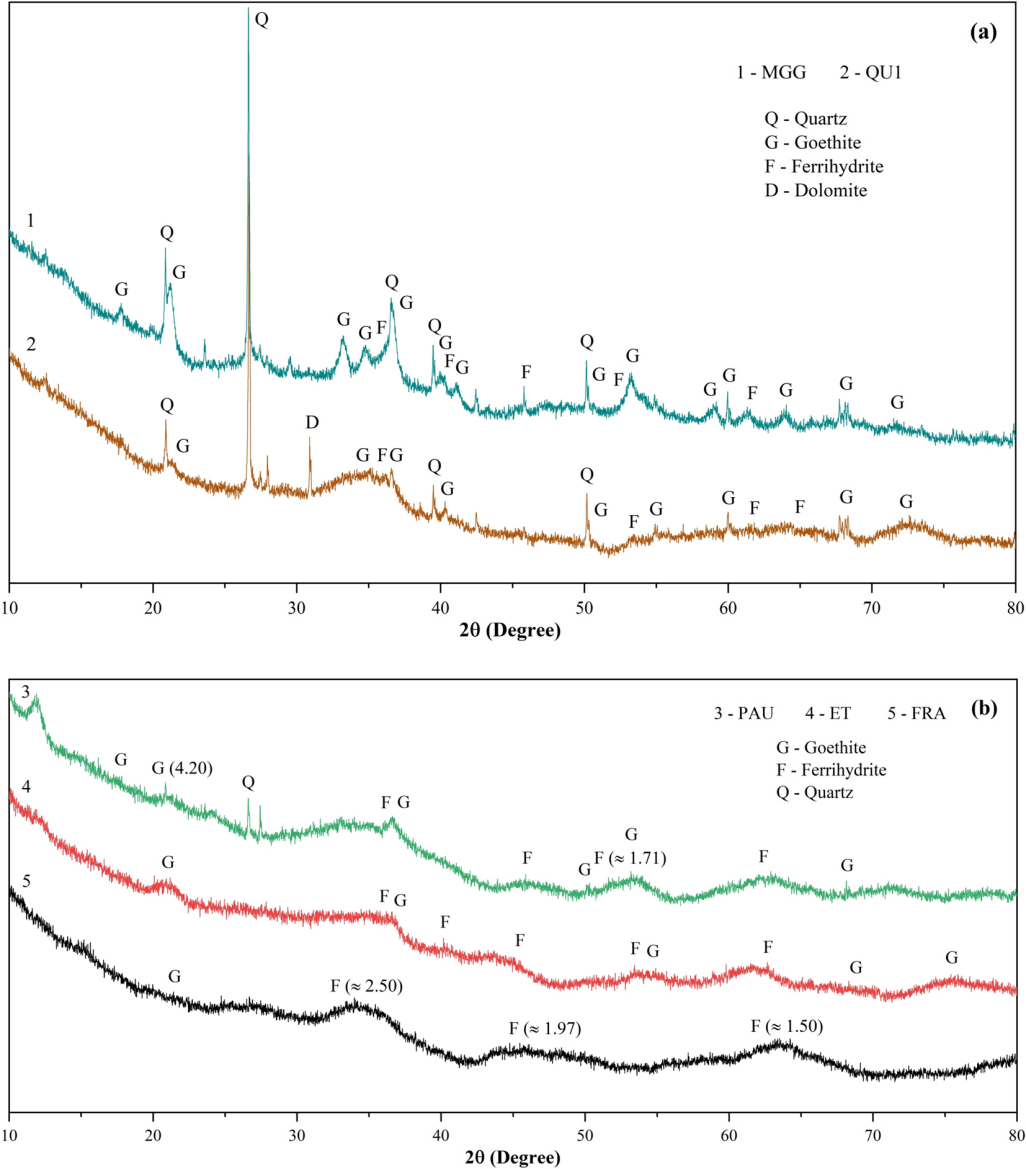
XRD diffraction patterns of ochreous precipitate samples from the adits (**a**) Rösche von Mit Gott gewagt (MGG) and Quelle Schacht 1 (QU1), (**b**) Pauline Stollen (PAU), Edeltraut Erbstollen (ET), and Franziska Erbstollen (FRA). G: goethite, F: ferrihydrite, D: dolomite, and Q: quartz (other XRD patterns are presented in Fig. S1)

Obtained XRD patterns are shown together with the peak positions of typical minerals in ochreous precipitate samples, which contain different phases (Figs. 6 and S1). The XRD results indicate that mine drainage Fe precipitates in the area are fine-grained and often poorly crystalline. Goethite and ferrihydrite were the dominant Fe precipitates and were the most common Fe oxides and hydroxide minerals found. Goethite was identified as the principal crystalline phase of Fe precipitates with intense peaks at 4.20 and 2.45 Å. The possible presence of ferrihydrite in the samples was indicated by broad peaks at 2.50, 1.97, and 1.50 Å (Fig. 6). Minor amounts of silicates (quartz), dolomite, and amorphous components containing trace elements (unnamed minerals) were also observed. A peak for dolomite was detected in the sample collected at Quelle Schacht 1 (Fig. 6). A few peaks of quartz with varied intense peaks were identified in several samples. Quartz was abundantly present in samples collected at Quelle Schacht 1, Rösche von Mit Gott gewagt, Rudolf Stollen, and Stollen von Braut at 4.25, 3.34, 2.45, and 1.82 Å, whereas it was completely absent in the samples collected at Edeltraut Erbstollen and Franziska Erbstollen. The color of precipitates was commonly strong brown (7.5YR 4/6, 5/6, and 5/8), with some samples being yellowish red (5YR 4/6, 5/6, and 5/8) to dark yellowish-brown (10YR 3/6 and 4/6), indicating the dominant iron minerals in the samples.

#### FTIR results (chemical functional groups)

**Fig. 7:**
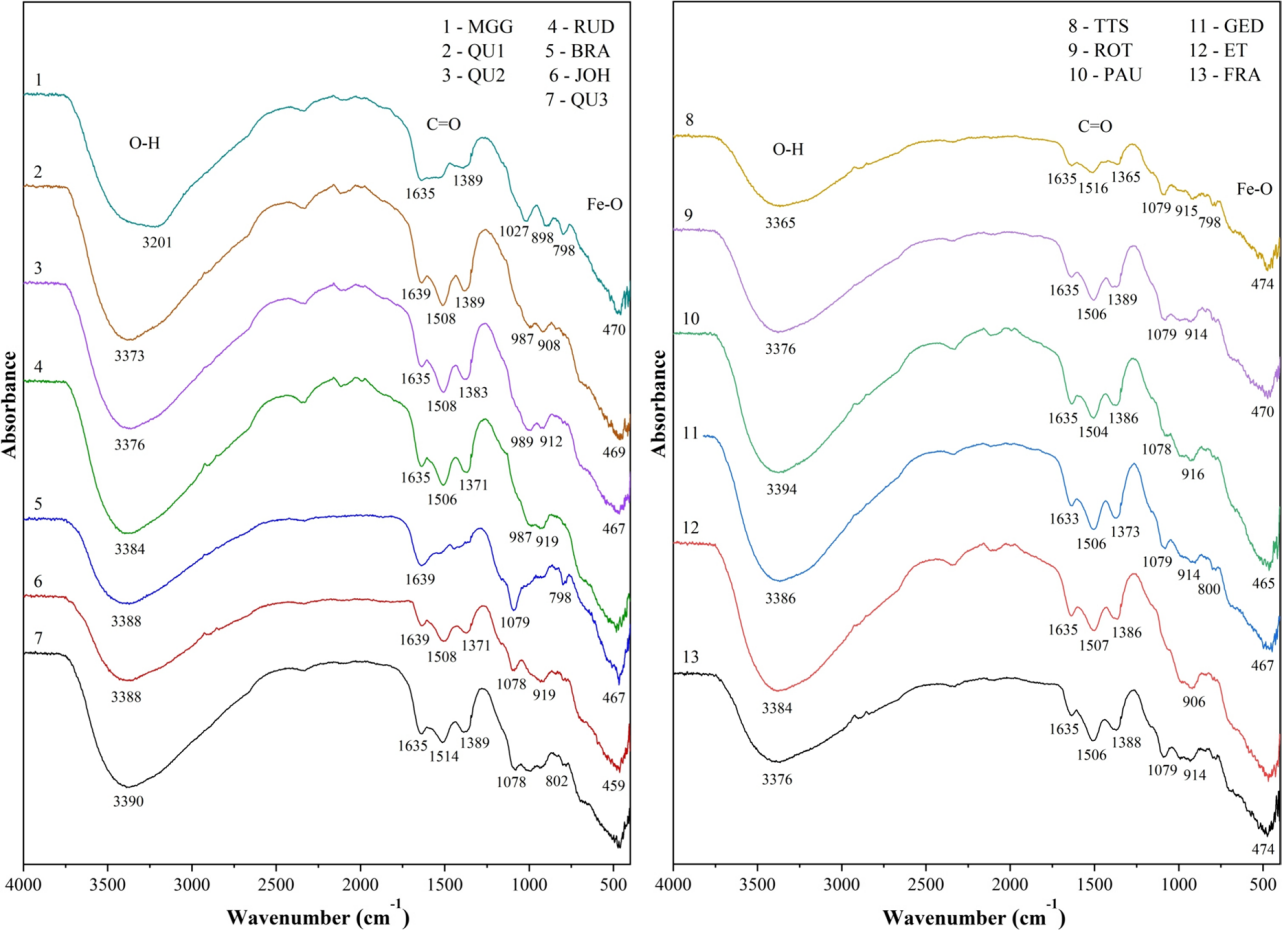
FTIR patterns of mine drainage ochreous samples showing adsorption band positions and characteristic peaks

The FTIR patterns of all precipitate samples show bands from adsorbed components (Fig. 7). One band of 3201 cm^−1^ was observed in the Rösche von Mit Gott gewagt sample, implying an O–H vibration in the goethite structure (Choo & Lee, 2002; Singh et al., 1999). The two characteristics of O–H deformations corresponding to the δ_OH_ and γ_OH_ bending vibrations were of goethite at ≈ 900 and ≈ 800 cm^−1^ (Gagliano et al., 2004; Schwertmann & Carlson, 2005; Peretyazhko et al., 2009; Equeenuddin et al., 2010; Bao et al., 2018). A Fe–O stretching was presented by a bending vibration at ≈ 470 cm^−1^. A broad O–H stretching band (ν_OH_) of structural hydroxyl groups with a vertex in all samples was observed with a wavenumber between 3650 and 3000 cm^−1^ (Fig. 7). The vibration was observed around a peak centered at about 3400 cm^−1^, corresponding to adsorbed water (Parikh et al., 2014), implying the presence of ferrihydrite minerals (Murad & Rojík, 2005). The absorbed carboxylic group was presented in the graph with the absorbance intensity of the FTIR spectrum band around 1400–1500 cm^−1^ (Fig. 7; Kumpulainen et al., 2007; Schwertmann & Fischer, 1973). All samples showed this band. The bands with a maximum of about 1635 cm^−1^ were considered as a bend of bonds in the H_2_O molecule (Parafiniuk & Siuda, 2006; Qiao et al., 2017). Ferrihydrite was observed with a band at about 1389 cm^−1^ and related to the adsorbed carboxylic group (Fig. 7).

#### Precipitate morphology

**Fig. 8:**
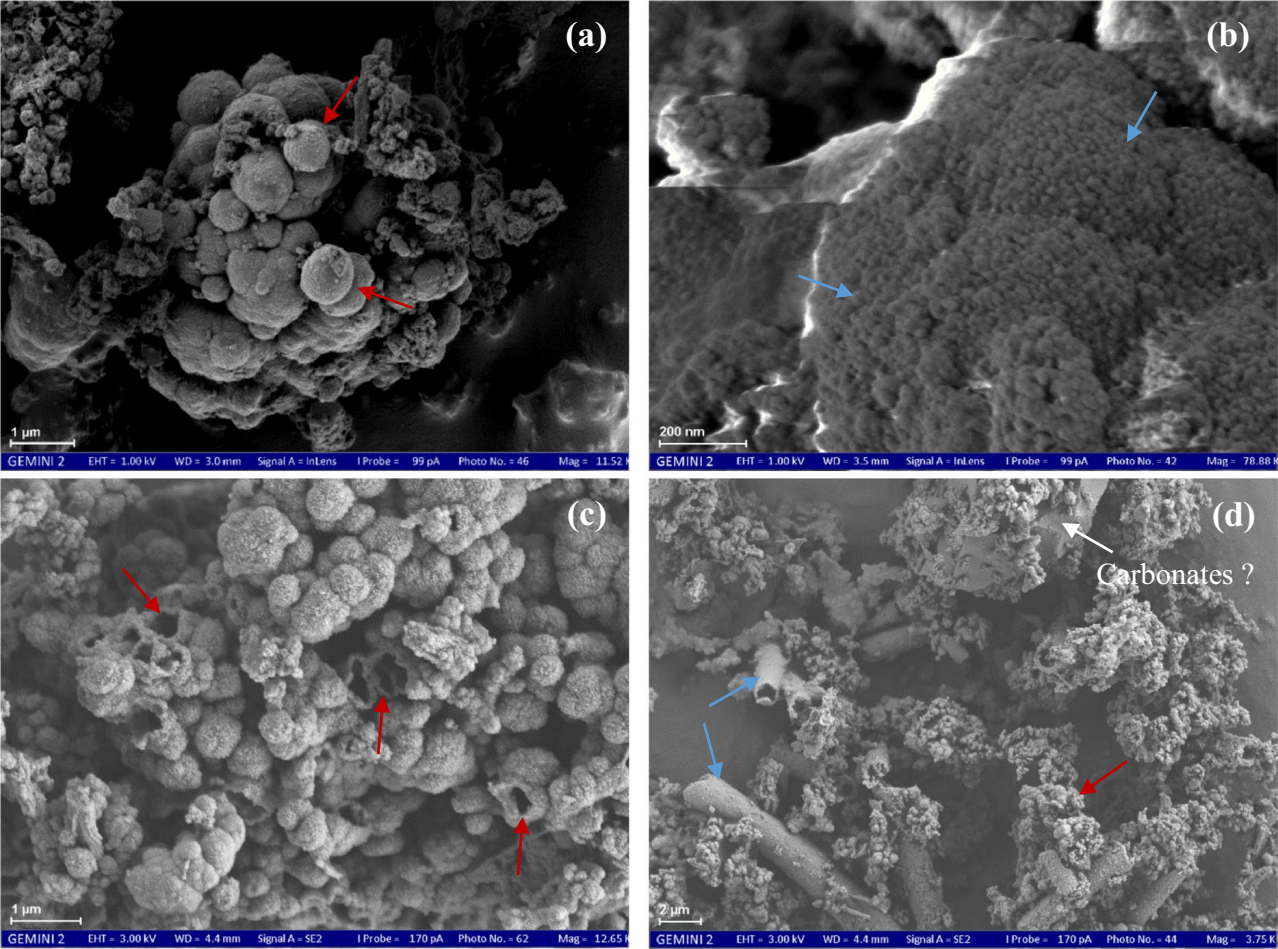
FE-SEM micrographs of precipitate samples on the drainage adit bottom in the area: (**a**) goethite, (**b**) ferrihydrite, (**c**) goethite (hollows inside), and (**d**) ferrihydrite (sheaths of bacteria) and goethite or amorphous Fe precipitate, corresponding to the different color arrows of goethite (dark red) and ferrihydrite (blue)

The observed goethite had a spherical form and rough morphological features with a size ranging from 0.5 to 1.0 µm (Fig. 8a; Gao & Schulze, 2010). Ferrihydrite was identified by highly aggregated spherical particles with a size of particles ranging from 13 to 30 nm (Equeenuddin et al., 2010; Fig. 8b). The iron oxyhydroxides were formed with fine-grained, poorly crystalline aggregates (ferrihydrite and goethite) and other occasionally observed mineral phases (Fig. 8d). Furthermore, FE-SEM images revealed the presence of clay minerals, such as montmorillonite, in the Stollen von Braut and Edeltraut Erbstollen samples (Fig. S2). This mineral was identified by its microstructure with a film-like appearance and thin flakes in the dried state under the FE-SEM. The long particle axis of this mineral is usually less than 1 to 2 μm. In this study, montmorillonite mostly showed particle edges rather than faces (Fig. S2; Velasco, 2013).

**Fig. 9:**
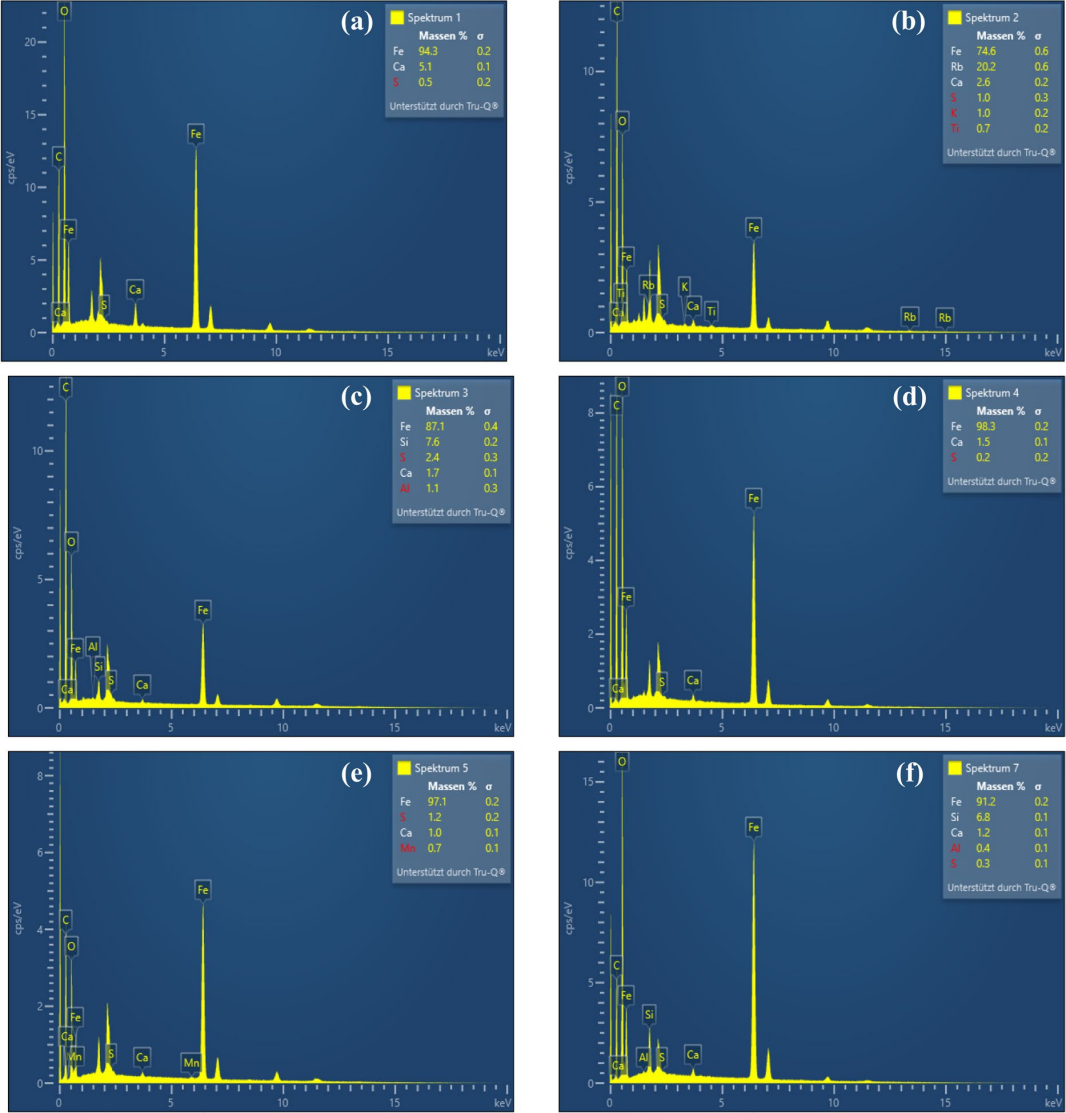
EDS analyses of ochreous samples (**a**) Pauline Stollen, (**b**) Roter Bach, (**c**) Rösche von Mit Gott gewagt, (**d**) Quelle Schacht 2, (**e**) Franziska Erbstollen, and (**f**) Quelle Schacht 1

The elemental composition of the samples was also determined semi-quantitatively using EDS analysis (Fig. 9). The elemental analysis indicated the presence of a wide variety of different elements observed in the samples, such as Fe, Mn, Ca, Al, C, O, Si, Ti, and S, with the predominant Fe phases. The samples were composed of largely accumulated Fe, and the content of Fe was in the range of 74.6–98.3%. The EDS results also showed other elements such as Ca (1.0–5.1%), Al (0.4–1.1%), Si (6.8–7.6%), S (0.2–2.4%), and Mn (0.7%) in some of the precipitates. In addition, Rb was detected in the Roter Bach, up to 20.2% (Fig. 9).

**Fig. 10:**
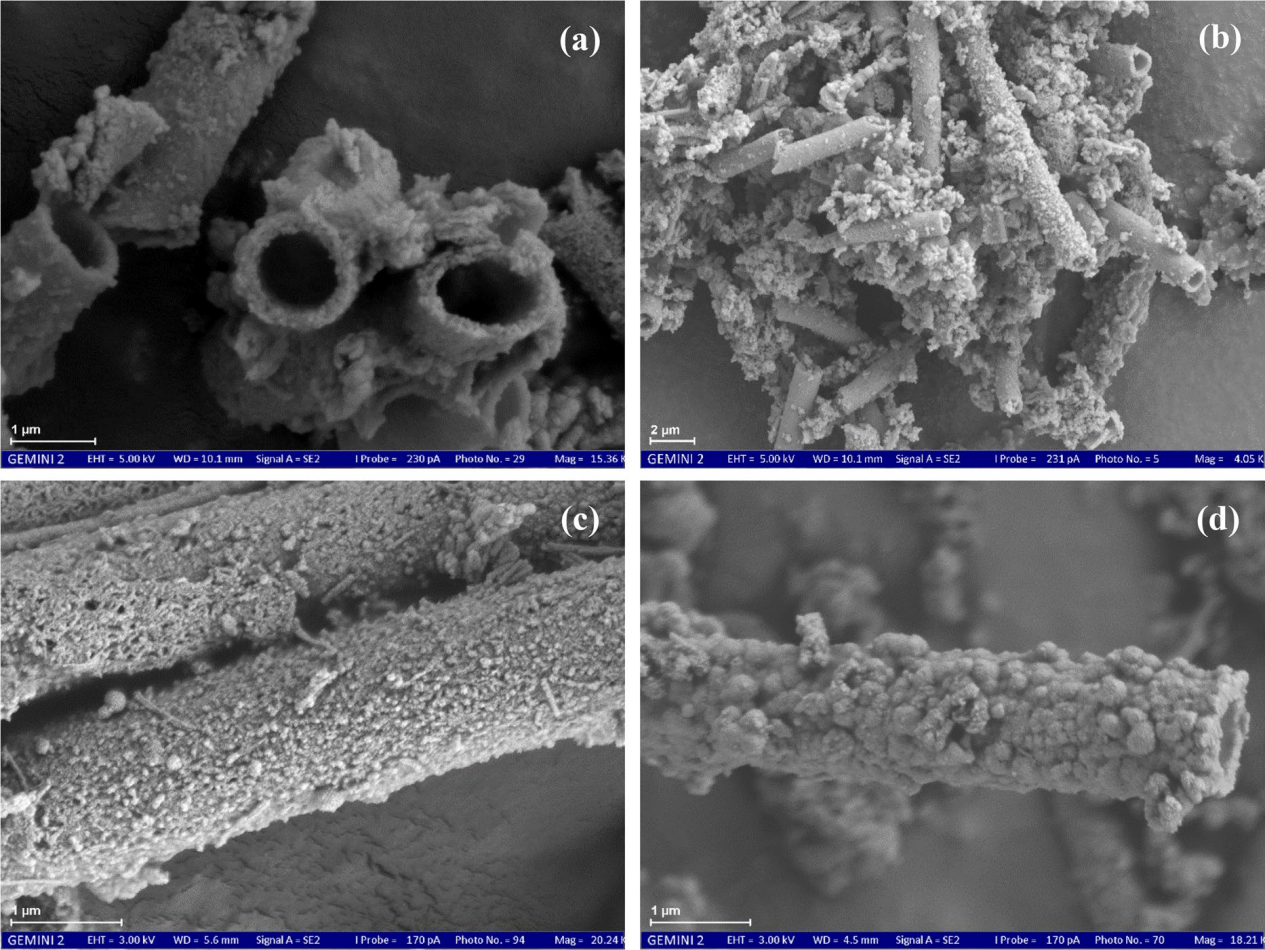
Structure and morphology of a *Leptothrix ochracea*, (**a**) broken *L. ochracea* sheaths and bodies, (**b**) smooth sheaths and random orientation, (**c**) *L. ochracea* sheaths with tiny particles, and (**d**) with large globular particles

Based on FE-SEM images, *Leptothrix ochracea*, one of the *Leptothrix* species of iron-oxidizing bacteria, was observed in the samples (Fig. 10; Kunoh et al., 2015; Plewka, 2020). This bacterium was observed ubiquitously in the precipitates at Quelle Schacht 1, Quelle Schacht 3, Roter Bach, Treue tiefer Stollen, Rudolf Stollen, Edeltraut Erbstollen, and St. Johannes Erbstollen sites. They excrete a hollow and tubular Fe(III)-oxyhydroxide encrusted sheath (Fleming et al., 2013; MacDonald et al., 2019) with a common diameter of about 1 μm (Fig. 10) and a length that can reach hundreds of micrometers (Baskar et al., 2012). A large number of tubular structures with shapes ranging from straight to slightly curved is observed, resembling sheaths of the iron-oxidizing bacteria *Leptothrix ochracea* (Baskar et al., 2012; Fig. 10b). Moreover, nano-scaled sub-spherical and irregularly shaped Fe-rich particles were attached to the bacterial sheaths. Three different levels of encrustation of the *Leptothrix* sheaths are detected in the precipitated samples (Fig. 10). They are organized into smooth sheaths (Fig. 10a, b) and are coated with tiny particles (Fig. 10c). Otherwise, the sheaths of *Leptothrix* were coated more heavily with larger particles (Fig. 10d).

### Oxygen and hydrogen stable isotopic characteristics

**Fig. 11:**
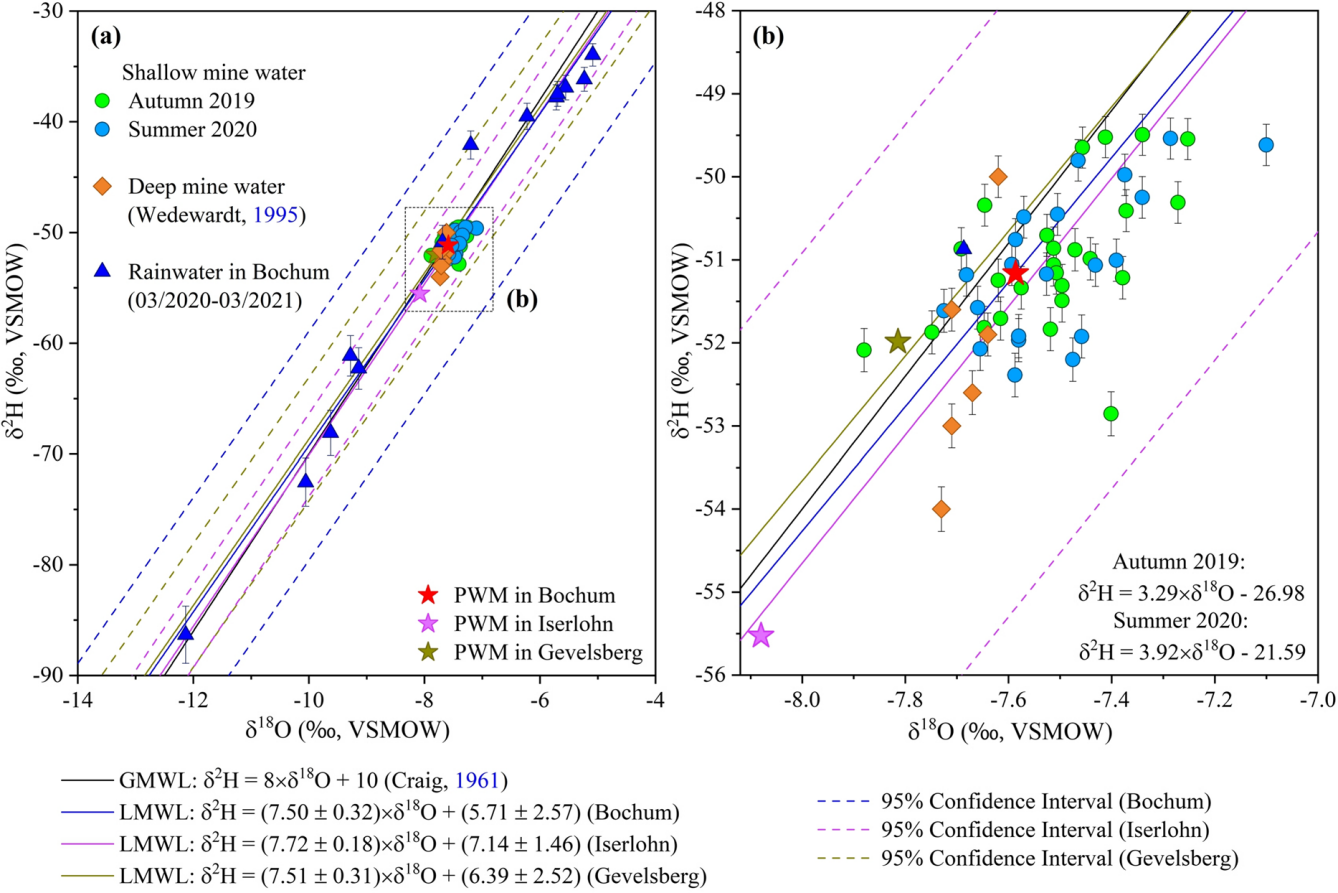
(**a**) Relationship between δ^18^O and δ^2^H values in shallow mine water for autumn (2019) and summer (2020), deep mine water, and rainwater samples compared to the GMWL and LMWLs with confidence intervals of isotopic data at Bochum, Iserlohn, and Gevelsberg, and (**b**) a close view of the mine water isotopic compositions. Stable isotopic values from mine water compared to the LMWLs and local precipitation-weighted means (PWM); error bars are within the size of the data points

**Fig. 12:**
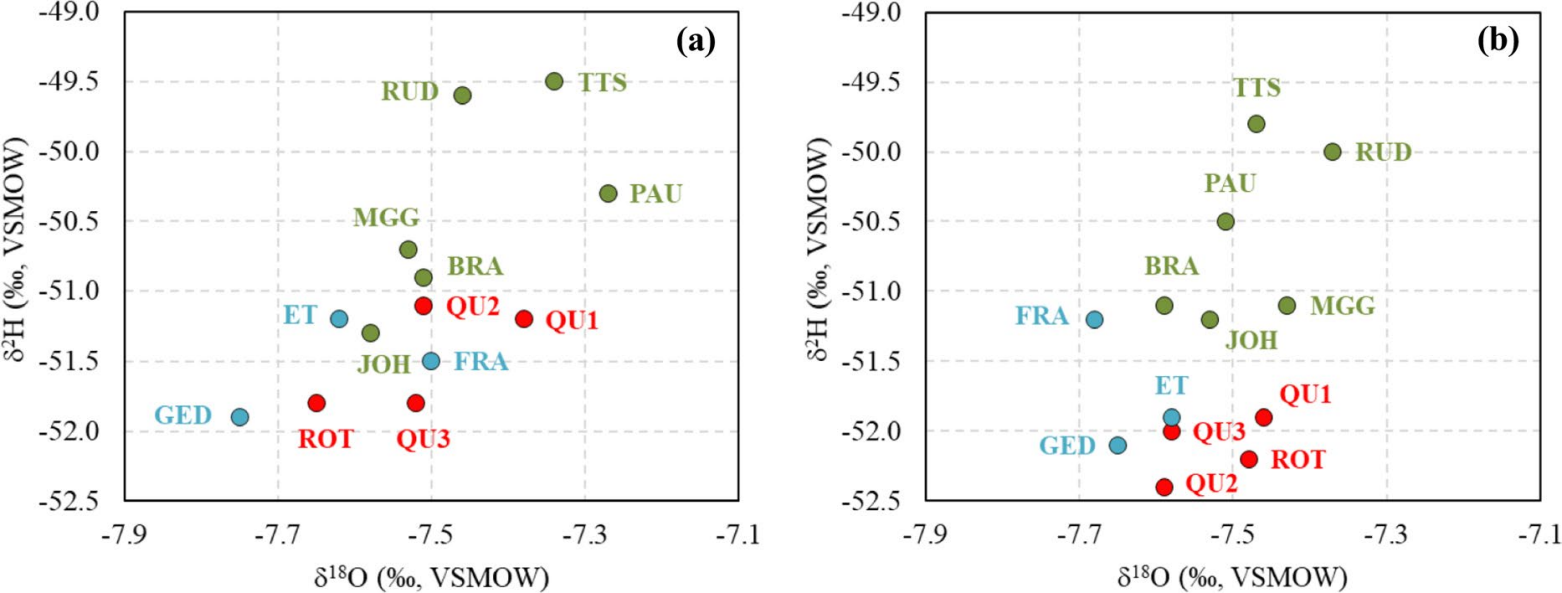
Relationship between δ^18^O and δ^2^H values in the three water groups, (**a**) in autumn 2019 and (**b**) in summer 2020

Oxygen (δ^18^O) and hydrogen (δ^2^H) stable isotopic analyses and calculated deuterium excess values (*d*-excess) are shown for collected mine water and rainwater samples (Tables S2, S3, and Fig. 11). In autumn 2019, the δ^18^O values of mine water samples ranged between − 7.88 and − 7.25 ‰, with an average value of − 7.51‰, and δ^2^H values ranged between − 52.9 and − 49.5 ‰, with a mean value of − 51.0 ‰ (*n* = 25). In summer 2020, these values varied from − 7.73 to − 7.10 ‰ (a mean of − 7.50 ‰) for δ^18^O and from − 52.4 to − 49.5 ‰ (a mean of − 51.0 ‰) for δ^2^H (*n* = 21) (Table S2). Calculated average *d*-excess values were 9.11 ‰ for autumn 2019 and 8.98 ‰ for summer 2020. δ^18^O and δ^2^H values of rainwater in this study ranged from − 12.13 to − 5.09‰ and − 86.3 to − 34.0‰ (*n* = 13), respectively. δ^18^O and δ^2^H mean values of rainwater were − 7.59 and − 51.2‰, respectively (Table S3). Stable isotopic compositions of samples were compared to the Global Meteoric Water Line (GMWL, δ^2^H = 8 × δ^18^O + 10) (Craig, 1961; Fig. 11). In addition, these values were compared to the isotopic signatures of local precipitation in the area. Three stable isotopic datasets of rainwater samples were used to derive the Local Meteoric Water Line (LMWL) of the region: (1) rainwater samples collected in Bochum during this study (Fig. 3), LMWL: δ^2^H = (7.50 ± 0.32) × δ^18^O + (5.71 ± 2.57) (*R*^2^ = 0.98, *n* = 13), (2) rainwater samples collected on the roof of the German Cave Museum Iserlohn (Dechen Cave), Iserlohn (March 2007–August 2013) with around 24 km distance to the current study area, LMWL: δ^2^H = (7.72 ± 0.18) × δ^18^O + (7.14 ± 1.46) (*R*^2^ = 0.98, *n* = 65) (Riechelmann et al., 2017), and (3) samples taken at Gevelsberg (December 2014–June 2017) with a 2.5-km distance to the south-west of the meteorological station Gevelsberg-Oberbröking and within the southern boundary of the study area, LMWL: δ^2^H = (7.51 ± 0.31) × δ^18^O + (6.39 ± 2.52) (*R*^2^ = 0.98, *n* = 28) (Riechelmann et al., 2022). These data were also plotted on the δ^18^O-δ^2^H diagram with the GMWL (Fig. 11). The datasets were used to calculate the weighted mean isotopic compositions for each location, e.g., Bochum (δ^18^O: − 7.59 and δ^2^H: − 51.2 ‰), Iserlohn (δ^18^O: − 8.08 and δ^2^H: − 55.5 ‰), and Gevelsberg (δ^18^O: − 7.81 and δ^2^H: − 52.0 ‰). Moreover, the stable isotopic data of deep mine water from Friedlicher Nachbar, Robert Müser, and Heinrich coal mines were added to this study for reference. The isotopic values of deep mine water ranged from − 7.73 to − 7.62 ‰ for δ^18^O and from − 54.0 to − 50.0‰ for δ^2^H (Table S2c; Wedewardt, 1995). In addition, a difference between the stable isotope compositions of three mine water groups was observed (Fig. 12).

## Discussion

### Interpretation of mine water chemistry and potential precipitate phases

At sampling locations, the major ion concentrations of the samples in group 3 were higher than samples in groups 1 and 2, implying the dissolution of minerals occurs strongly. The high HCO_3_^−^ concentration reflected the dissolution of carbonate-rich minerals and a strong buffering capacity in the host rock in the study area. Common minerals found in the host rocks include calcite, feldspar, quartz, mica, and iron oxide minerals (Wisotzky, 2018). Wedewardt verified the occurrence of carbonate minerals in rocks from the Upper Carboniferous strata in the area, with carbonate contents in sandstone and sandy shales ranging between 0.22 and 1.83 wt% (Wedewardt, 1995). Furthermore, substantial concentrations of HCO_3_^−^ in the drainage water can result from the chemical weathering of rock-forming minerals (Younger et al., 2002; Huang et al., 2018). By alkalizing the environment and consuming protons, it usually causes dissolved CO_2_(*aq*) to convert to HCO_3_^−^ and raises the concentration of dissolved carbonate species. The consumed dissolved CO_2_(*aq*) is replenished from the atmosphere and the decomposed organic materials in the surface area. For instance, the weathering of anorthite which exists in the area is as follows:

$$\text{Anorthite}+4 {\text{H}}_{2}\text{O}+2 {\text{CO}}_{2}(aq)=2 {\text{HCO}}_{3}^{-}+{\text{Ca}}^{2+}+2\text{ Gibbsite}+2 {\text{SiO}}_{2}(aq)$$

In the study area, Fe concentrations in water samples were higher than Mn concentrations. According to Singer and Stumm (1970), dissolved Fe was produced by the oxidative dissolution of disulfides. In this area, Schöpel (2019a) also indicates that dissolved Fe in the drainage water originated from the oxidative dissolution of pyrite. However, Fe concentration was relatively low in water samples, implying that most dissolved Fe precipitates through oxidation and hydrolysis reactions (Singh et al., 1999; Winland et al., 1991). In the study area, according to our observations, precipitates coating the flowing bottoms of drainage adits have different colors, which might indicate the presence of iron phases. The mine water from the study area is similar in composition to mine water from the Upper Silesian coal basin in Poland (Janson et al., 2009, 2016), while it differs from the mine waters from Derbyshire, Yorkshire, and Durham coalfields in the UK (Banks et al., 1997; Jarvis & Younger, 1997).

The saturation indices were calculated to assess mineral stability and theoretically identify secondary minerals that might precipitate from mine water, especially the Fe and Mn precipitates. In this study, geochemical equilibrium was assumed for precipitate mineral phases. Based on obtained SI results, Fe phases could possibly be precipitated, especially minerals such as goethite and ferrihydrite. Although the SI values for hematite and magnetite exceed + 0.2, these minerals are rarely precipitated in the surface environment due to kinetic hindrance (Lee & Chon, 2006; Merkel & Planer-Friedrich, 2005). In the study area, under circumneutral pH and low SO_4_^2−^ concentration conditions, jarosites are not formed from collected water samples (Table S1). This is also confirmed by the fact that water samples were undersaturated with respect to all jarosite minerals. In addition, carbonate minerals might be precipitated because the SI values are greater than zero or in equilibrium in a few water samples (Table S1). XRD and FE-SEM analyses of precipitate samples identified these minerals (discussed in the below section).

Furthermore, co-precipitation can occur by precipitating hydrous ferric oxides, ferric minerals, and manganese carbonate. Precipitation of Mn in carbonate minerals was observed in mining environments with high alkalinity (Cidu, 2011). PHREEQC modeling supported this mechanism since mostly Fe phases and rhodochrosite (MnCO_3_) had a trend of SI values approaching zero. Overall, the formation of Fe and Mn precipitations can lead to a decrease in minor element concentrations in mine water. Precipitates were confirmed by PHREEQC modeling; they are consistent with the mineralogical analyses and are discussed below in more detail.

### Ochreous precipitate compositions and minor element binding

The XRD analysis showed the presence of crystallized phases and identified crystalline and amorphous minerals in the precipitate samples in the area. According to Bigham et al. (1992), Dold (2003), and Schroth and Parnell (2005), the presence of secondary Fe minerals with weak peaks can only be differentiated through a detailed examination of low-intensity peaks. Due to ferrihydrite having poorer crystallinity characteristics than goethite, goethite peaks are more clearly visible (Fig. 6). Schwertmannite is detected and stable in low pH environments (Gao & Schulze, 2010). Thus, in this study with samples of high pH (6.6–7.0), schwertmannite is not formed from drainage mines and is most likely not present in the precipitate samples. However, although in some samples (e.g., Roter Bach and Edeltraut Erbstollen sites), the shape of a few particles is similar to schwertmannite, and its presence is suggested by FE-SEM analysis, additional methods would be required to verify the presence of this mineral.

In this study, the precipitate samples were mixtures of iron oxides, oxyhydroxides, and other minerals like quartz. For example, the Edeltraut Erbstollen sample consists mainly of goethite and poorly crystallized ferrihydrite. Most samples consist predominantly of goethite and ferrihydrite with a lesser amount of quartz (e.g., Pauline Stollen sample) (Fig. 6). The predominance of goethite and ferrihydrite or mixtures of both in the samples can be explained by environmental conditions. Ferrihydrite is commonly precipitated from mine drainage at a higher pH value. At mine drainage sites, when the pH value of the water exceeds 6.5, the minerals are composed of ferrihydrite or a mixture of ferrihydrite and goethite in the precipitates formed (Park et al., 2018). Moreover, goethite is more stable and less soluble than ferrihydrite. This mineral tends to be supersaturated in low-temperature solutions (Alpers & Nordstrom, 1999; Hammarstrom et al., 2005). As mentioned above, in the study area, mine water had pH values of 6.6–7.0 and low water temperature (Table 1). Therefore, comparing the mine water conditions in the area, these are also in agreement with previous studies. In addition, the presence of quartz in the samples is most probably a product of the weathering process because the geology of the area consists of siliciclastic materials (sandstone and siltstone). In addition, the associated variation in color of ochreous precipitates from strong brown, yellowish red, to dark yellowish-brown can serve as an indication of the presence of iron oxidation products in the environment in which precipitates form.

The obtained FTIR patterns support the identification of ferric oxyhydroxides and the detection of changes in the reactive functional groups and mineral phases (Jin et al., 2020). The FTIR spectra of all samples do not show a substantial difference among samples, indicating the similarity of minerals in the samples. The 10 out of 13 samples presented a similar spectrum and only a slight difference between the peaks of Rösche von Mit Gott gewagt, Stollen von Braut, and Treue tiefer Stollen samples compared to other samples, especially the O–H stretching vibration (Fig. 7). According to the obtained FTIR spectra, the vibrational bands in the goethite are considerable. This supported the XRD analysis in the detection of goethite in the samples. The presence of ferrihydrite at 1389 cm^−1^ is in agreement with previous studies that found ferrihydrite formation was promoted in areas with high organic carbon content (Equeenuddin et al., 2010; Kumpulainen et al., 2007; Schwertmann & Fischer, 1973). Consequently, the FTIR analysis of the samples also supported the XRD results.

The FE-SEM/EDS analyses allowed the verification of the XRD patterns and aided in the identification of poorly crystalline minerals. Goethite is characterized by spherical goethite morphological features (Fig. 8a). This is in good agreement with an explanation of the shape of goethite via FE-SEM images as reported by Gao and Schulze (2010). In addition, the goethite particles showed hollows inside, implying the transformation between mineral phases (Fig. 8d; Peretyazhko et al., 2009). When the sulfate content is low, goethite can co-precipitate with other weakly crystalline Fe minerals (Gao & Schulze, 2010). The FE-SEM images also confirmed and observed this co-precipitation (Fig. 8). Moreover, carbonate minerals might be present in the precipitates (Fig. 8d). However, additional methods should be applied to verify this finding.

The elemental composition of the ochreous samples, especially showing the presence of Fe oxyhydroxide phases, was assessed. Although the samples have been well homogenized, EDS analysis is not representative of the entire components of one sample because it is performed exclusively at points on the sample surface. According to EDS analysis, Rb was only identified in one sample (Fig. 9). The presence of Rb in the Roter Bach sample might be originating from the chemical weathering of Illite, K-feldspar, and mica from the host rocks in the area (Grobe & Machel, 2002). Thus, Rb in this sample is a contamination element and it is not representative of all samples. The appearance of Si and O might be due to the clay or quartz minerals in the samples. This was also supported by the detection of quartz minerals in XRD analyses (Fig. 6) and clay minerals at the Stollen von Braut and Edeltraut Erbstollen sites (Fig. S2). Moreover, looking at the elemental composition of the clay mineral (montmorillonite) which was observed in these two samples, Al and Si coexisted with Fe precipitates in the samples. The presence of Al in the precipitates (see Fig. 9) indicates that it might come from clay minerals (Fig. S2), accumulated and mixed with Fe precipitates in the collected samples. In addition, due to low Al concentration in water samples (< 0.01 mg L^−1^) and high pH values of mine waters in this study (pH 6.6–7.0), Fe and Al hydrolyze and precipitate as hydroxide or hydroxyl-sulfate compounds, and Al is mostly removed from the water (Kim & Kim, 2004). The presence of C and O might indicate the existence of organic matters that provide a favorable environment for the precipitation of ferrihydrite (Bigham et al., 1992; Kumpulainen et al., 2007). In addition, the values of molar Fe/S ratios were calculated and ranged between 36.3 and 492 (Fig. 9), indicating the existence of goethite and ferrihydrite in the samples (Equeenuddin et al., 2010). The presence of Mn, Si, and Ti in the samples found by EDS analysis can rather be explained by a co-precipitation process with the Fe precipitates. In addition, Ca was also detected by EDS analysis in the samples, and this is in good agreement with PHREEQC results (Table S1). Overall, the obtained results from mineral analyses are in agreement with the predicted saturation of Fe phases in the samples by PHREEQC.

The presence of *Leptothrix ochracea* in the precipitates indicated their cooperative role in the iron oxidation process and in controlling dissolved Fe speciation in mine water. It forms in circumneutral water of drainage ditches, springs, and lakes, and it is common in a circumneutral pH environment with a source of reduced Fe minerals (Kunoh et al., 2015), as well as in an environment with an abundance of Fe(II) (MacDonald et al., 2018). The hypothesis of biologically induced precipitation of ferric oxyhydroxides is supported by the occurrence of iron bacteria in mine water (Máša et al., 2012). In the area, the iron oxidation process occurs when mine water circulates in abandoned coal mines. Micro-organisms (chemosynthetic microbes) that live in the mine water environment oxidize bivalent Fe(II) to trivalent Fe(III) to obtain their vital energy. They play an important role in iron biogeochemical cycling, as well as increased crystallization and absorption of Fe and other elements (Máša et al., 2012). The sheath of this bacterium was composed primarily of poorly crystalline iron oxides.

### Water sources — δ^18^O and δ^2^H values of water samples

The oxygen and hydrogen isotope values of rainwater samples collected in Bochum during this study result in a LMWL of δ^2^H = (7.50 ± 0.32) × δ^18^O + (5.71 ± 2.57), which agrees with the LMWL’s of other rainwater collection sites in the region (Riechelmann et al., 2017, 2022). Furthermore, the LMWL of the study site corresponds with the LMWL’s of the GNIP (Global Network of Isotopes in Precipitation) stations Bad Salzuflen (δ^2^H = 7.72 × δ^18^O + 5.7) and Emmerich (δ^2^H = 7.66 × δ^18^O + 4.8; both 1978–2009, Stumpp et al., 2014), which are closest to the study area. Thus, the source region of precipitation for the study area and other collection sites in NW Germany is similar and is the result of the west–east horizontal transport of the atmospheric water vapor over Central Europe (Rozanski et al., 1982). Moreover, the LMWL slope (7.50) of the rainwater samples collected in this study is close to that of the GMWL (8; Craig, 1961; Rozanski et al., 1993), indicating no significant evaporation of the collected rainwater samples (Gat et al., 2000). A previous study confirms that the evaporation effect is negligible in the region (Riechelmann et al., 2017).

The precipitation-weighted mean (PWM) oxygen and hydrogen isotope values of rainwater collected in Bochum and Gevelsberg are alike, whereas the PWM δ^18^O and δ^2^H values of rainwater from the Iserlohn collection site are significantly lower (0.49 ‰ for δ^18^O and 4.30 ‰ for δ^2^H) (Fig. 11). These differences in the PWM oxygen and hydrogen isotope values of the different collection sites can be best explained by dissimilar (i) local parameters such as altitude and (ii) time period of collection. The collection site in Bochum displays the lowest altitude (94 m a.s.l.), whereas the rainwater collection sites in Gevelsberg and Iserlohn were located at higher altitudes of 206 m a.s.l. and 175 m a.s.l., respectively (Riechelmann et al., 2017, 2022). According to Sharp (2007), the δ^18^O value of precipitation decreases on average by around 0.26‰ per 100 m for elevations up to 5000 m. The altitude differences between Bochum, Gevelsberg, and Iserlohn rainwater collection sites are 112 m and 81 m. Consequently, PWM δ^18^O and δ^2^H values can be expected to be slightly lower for rainwater collected in Gevelsberg and Iserlohn than for rainwater collected in Bochum. Nevertheless, the difference of 4.30‰ for hydrogen isotope values and 0.49‰ for oxygen isotope values between the PMW of Bochum and Iserlohn is too big to be explained by altitude alone. The main difference between the three collection sites is the time period of collection (Bochum: March 2020–March 2021; Gevelsberg: December 2014–June 2017; Iserlohn: March 2007–August 2013). Due to the extreme year 2010, the PMW δ^18^O and δ^2^H values of rainwater collected in Iserlohn are shifted to more negative values. This year is characterized by a lower mean annual temperature (7.1 °C) than the other years of the sampling period (9.6–11.3 °C; Riechelmann et al., 2017). Temperature is the main driving force for δ^18^O and δ^2^H values variability in precipitation in this region (e.g., Riechelmann et al., 2017, 2022; Rozanski et al., 1982, 1993). The mean annual temperature for the rainwater collection period in Bochum is 10.5 °C (Rudolf Geiger climate station), similar to previously reported mean annual temperatures (Riechelmann et al., 2017, 2022). Hence, it can be assumed that δ^18^O and δ^2^H of rainwater collected in Bochum are representative of the region, although it was only collected for one year.

The oxygen and hydrogen stable isotopes of groundwater are considered natural tracers to identify the origin of groundwater (Clark & Fritz, 1997; Vermeulen et al., 2014). At low temperatures, these isotopic compositions in groundwater do not change as a result of geochemical reactions in normal aquifers (Hoefs, 1997). Thus, it reflects the history and origin of groundwater. If groundwater is infiltrated directly by precipitation, it has the isotopic signature of precipitation (Liu et al., 2016), and the isotopic values are close to the mean values of rainwater (Clark & Fritz, 1997). The δ^18^O and δ^2^H values of mine water samples collected in two seasons (autumn 2019 and summer 2020) during this study plot on and close to the GMWL and LMWLs (Fig. 11), indicating the recharge source of mine water originated from present-day atmospheric precipitation. Furthermore, the oxygen and hydrogen isotope values of mine water samples scatter around the precipitation-weighted mean (PWM) oxygen and hydrogen isotope values of rainwater in Bochum and are close to the PWM δ^18^O and δ^2^H values of rainwater collected in Gevelsberg (star symbols in Figs. 11a, b). Thus, δ^18^O and δ^2^H values of mine water reflect the annual mean of local precipitation (Figs. 11 and S3) and do not show evaporation effects (Gat et al., 2000). Consequently, precipitation from all seasons infiltrating the ground is well-mixed and contributes to groundwater formation in the region as suggested by previous studies for European groundwaters (e.g., Rozanski et al., 1982). The δ^18^O and δ^2^H values of mine waters collected at the same adit show similar values within the measurement error for both collection periods (autumn 2019 and summer 2020; Fig. S3) supporting the interpretation above. Furthermore, it can be assumed that the transfer time of precipitation from surface to mine adit is longer than one year (residence time within the recharge environment; Clark & Fritz, 1997) to be able to reflect the annual mean oxygen and hydrogen isotope values of precipitation. Thus, mine water oxygen and hydrogen isotope values (autumn 2019 and summer 2020) cannot reflect the annual mean of precipitation collected during this study (March 2020 to March 2021) due to the time offset in the collection, but it reflects that of precipitation infiltrated during previous years.

Although the shallow mine water studied here is recharged by local precipitation, deeper water inside an aquifer may have differing oxygen and hydrogen isotopic signatures due to another source recharging the deeper mine water. This can be recognized from values that deviate from the local LMWL; thus, deeper mine water could originate from other sources (Varsányi et al., 2015). The oxygen and hydrogen isotopic compositions of deep mine water at 3 closed coal mines (Friedlicher Nachbar, Robert Müser, and Heinrich; Wedewardt, 1995) from the same area as the current study are set into context with those of shallow mine water collected in this study. The sampling depths of the deep mine water ranged between − 60 and − 458 (m a.s.l.) (Wedewardt, 1995). Some of the deep mine water samples display a little bit lower δ^2^H values than the shallow mine water from this study, whereas the δ^18^O values are similar (Fig. 11b). Nevertheless, deep mine water δ^18^O and δ^2^H values are close to the PWM of Bochum, Gevelsberg and Iserlohn (Fig. 11). Thus, both deep mine water and shallow mine water are recharged by local precipitation in the Ruhr area. However, it has to be kept in mind that the oxygen and hydrogen isotope values of deep mine water were obtained more than 25 years ago (Wedewardt, 1995) and local precipitation might have differed in its isotopic composition from today’s. This is supported by long-term GNIP stations’ δ^18^O, δ^2^H, and temperature data in Germany (covering up to 36 years). Oxygen and hydrogen isotope values of precipitation show an increase with increasing temperature over time due to climate change (Stumpp et al., 2014), which is an explanation for the slightly lower δ^2^H values observed for some of the deep mine waters (Wedewardt, 1995). A collection of local precipitation and shallow and deep mine waters in the same time interval is suggested for future studies to remove the last uncertainties. Moreover, the slight difference in isotopic composition of the mine water groups indicates a different sample collection period (autumn 2019 and summer 2020). The stable isotopes of group 1 are located on the upper side (green points), with higher stable isotope compositions compared to mine waters in groups 2 (blue) and 3 (red) (Fig. 12). Otherwise, these results also reflect the distinct existing conditions of these water groups. The concentrations of NO_3_^−^ and O_2_ in group 1 were the highest, followed by those in group 2, while NO_3_^−^ concentration was not detected in mine water in group 3, and the measured O_2_ content of mine water in this group was the lowest.

### Effects of iron precipitates on Ruhr River water

In the southern Ruhr area, where Upper Carboniferous formations are exposed, the formation mechanism of mine water may be explained by rainwater infiltration. Rainwater infiltrates from the surface of abandoned coal mines through the materials of the host rocks. Disulfide minerals in coals and related rocks are oxidized, releasing iron-rich, acidic solutions with potentially high trace metal concentrations (Wolkersdorfer, 2008). The released Fe(II) can be transported and further oxidized to Fe(III), as documented by brownish-reddish precipitates along mine drainage adits and the entrance of adits (Fig. 2). Simultaneously, the dissolution of carbonates and silicates from the rocks may change the chemical composition of mine water. The observed consequence is an accumulation of secondary iron minerals. Due to the precipitation of ferric oxyhydroxides along the length of the adits, Fe concentrations in discharged mine water are low. Trace elements might be adsorbed and/or co-precipitated by the ferric oxyhydroxides.

In our recent study, implications of climate change related to mine drainage from abandoned coal mines in the study area were monitored and investigated from 2018 to 2019 (Tran et al., 2022). Mine water discharges with low concentrations of trace elements from the drainage adits and drains directly to the receiving water resources, e.g., the river Ruhr. In addition, the mine water flows are much smaller than the receiving water course. For example, a maximum mine water flow rate of about 500 L s^−1^ (0.5 m^3^ s^−1^) was measured at Schlebuscher Erbstollen adit (Schöpel, 2019b), while the smallest discharge in the Ruhr River was recorded at the Hattingen/Ruhr gauging station with *Q* = 16.3 and 14.8 m^3^ s^−1^ in 2018 and 2020, respectively (AWWR, 2018, 2020). Therefore, the effects of mine water on stream water quality are substantially weakened by dilution. Moreover, it does not consider metal remobilization by pH lowering a realistic scenario because HCO_3_^−^ concentration is always the most dominant ion and mine water becomes more oxidizing in the presence of O_2_, Fe, and Mn, which tend to precipitate as solid phases. Compared to the permissible limits (e.g., the EU Direction, European Union, 2020), harmful compositions (e.g., Fe and Mn) should be addressed further to use mine water.

## Conclusions

This study was carried out with a multi-method approach to provide insights into understanding the characteristics of the mine water chemistry, mineral composition of ochreous precipitates, recharge source of mine water, and effects of iron precipitates on Ruhr River water in the southern Ruhr area. At the mine drainage locations where ochreous precipitates are present, chemical analyses indicated that mine water was circumneutral, with pH ranging between 6.6 and 7.0, and the dominance of HCO_3_^−^, SO_4_^2−^, Ca^2+^, and Na^+^ in the water. The maximum Fe and Mn concentrations were 5.2 and 1.1 mg L^−1^, respectively. Saturation indices suggested the precipitation of secondary mineral phases. The mine water was saturated generally with respect to goethite, ferrihydrite, and some other phases. Obtained XRD data and FE-SEM images indicated that the ochreous mineral assemblages precipitated from the mine drainage are mainly composed of goethite and ferrihydrite, which were the dominant Fe-oxyhydroxide minerals, followed by lower amounts of quartz, dolomite, and clay minerals. Simultaneously, the presence of several metals showed their co-existence in the precipitates. The bacteria in the precipitates of secondary minerals were investigated with *Leptothrix ochracea* found in the precipitate samples, and they had an important role in the Fe(II) oxidation and crystallization of Fe phases. In addition, the δ^18^O and δ^2^H values of mine water samples were plotted on the GMWL and LMWLs, suggesting that the dominant water source from abandoned coal mines was recharged locally by modern rainfall. The evaporation effect and seasonal changes were not observable in this study. About half of mine drainage adits show ochreous deposits covering the streambed, which indicates the environmental relevance of the southern Ruhr area. Future work may examine the seasonal change of mineral precipitates from the abandoned coal mine drainage, as well as analyze the S-isotopic composition of SO_4_^2−^ in mine water to clarify sulfide mineral oxidation in the study area. To assess the mobility of dissolved metals in mine water, the concentration of trace elements from ochreous precipitates should be analyzed in future research. This study was conducted to verify the characteristics of mine water settings as a tool for the assessment of environmental relevance, and the obtained results might support effective management of water use.

### Supplementary Information

Below is the link to the electronic supplementary material.

Supplementary file1 (DOCX 1860 KB)

## Data Availability

The stable isotope data of deep mine water (provided in Supplementary information, Table S2c) supporting the findings of this study are available at the Library of Ruhr-University Bochum. Example from: https://hbz-ubo.primo.exlibrisgroup.com/permalink/49HBZ_UBO/mnkbqv/alma991014872089706471.
