# Supplementary material for: Environmental relevance monitoring and assessment of ochreous precipitates, hydrochemistry and water sources from abandoned coal mine drainage
Source: Environ Monit Assess. 2024 Jul 4;196(8):700. doi: 10.1007/s10661-024-12858-x (PMC11224094; doi:10.1007/s10661-024-12858-x)
Supplement: Supplementary file 1 — Supplementary file1 (DOCX 1860 KB) [file 10661_2024_12858_MOESM1_ESM.docx]

**Environmental relevance monitoring and assessment of ochreous precipitates, hydrochemistry and water sources from abandoned coal mine drainage**

*Environmental Monitoring and Assessment*

Tuan Quang Tran ^1, 2, *^

^1^ Department of Hydrogeochemistry and Hydrogeology, Institute of Geology, Mineralogy and Geophysics, Faculty of Geosciences, Ruhr-University Bochum, Universitätsstraße 150, 44801 Bochum, Germany

^2^ Faculty of Geosciences and Geoengineering, Hanoi University of Mining and Geology, No. 18, Pho Vien, Duc Thang Ward, Bac Tu Liem District, Hanoi, Vietnam

quang.tran@rub.de; tranquangtuan@humg.edu.vn; ORCID code: 0000-0001-9614-126X

Sylvia Riechelmann ^1^

^1^ Department of Sediment and Isotope Geology, Institute of Geology, Mineralogy and Geophysics, Faculty of Geosciences, Ruhr-University Bochum, Universitätsstraße 150, 44801 Bochum, Germany

ORCID code: 0000-0002-8384-9661

Andre Banning ^3^

^3^ Department of Applied Geology, Institute of Geography and Geology, University of Greifswald, Friedrich-Ludwig-Jahn-Str. 17A, 17489 Greifswald, Germany

ORCID code: 0000-0002-7593-9122

Stefan Wohnlich ^1^

^1^ Department of Hydrogeochemistry and Hydrogeology, Institute of Geology, Mineralogy and Geophysics, Faculty of Geosciences, Ruhr-University Bochum, Universitätsstraße 150, 44801 Bochum, Germany

* Corresponding author: Tuan Quang Tran (quang.tran@rub.de; tranquangtuan@humg.edu.vn)

**Table S1** Selected mineral phases saturation indices for ochreous precipitates from drainage adits computed using PHREEQC

| Phase | Chemical formula | Saturation Indices (SI) | | | | | | | | | | | | |
| --- | --- | --- | --- | --- | --- | --- | --- | --- | --- | --- | --- | --- | --- | --- |
|  |  | QU1 | QU2 | QU3 | ROT | MGG | TTS | PAU | RUD | BRA | ET | GED | JOH | FRA |
| **Iron** |  |  |  |  |  |  |  |  |  |  |  |  |  |  |
| Fe(OH)_3_(a) | Fe(OH)_3_ | 1.23 | 0.34 | 1.32 | 0.27 | 1.19 | 0.69 | 1.43 | 1.19 | 2.10 | 1.26 | 1.44 | 1.32 | 1.03 |
| Fe_3_(OH)_8_ | Fe_3_(OH)_8_ | 0.89 | -0.95 | 1.40 | -1.13 | 0.97 | -0.70 | 1.07 | 0.92 | 2.33 | 0.75 | 1.94 | 0.98 | 0.66 |
| Ferrihydrite | Fe_2_O_3_.9H_2_O | 7.94 | 6.16 | 8.12 | 6.01 | 7.86 | 6.87 | 8.35 | 7.86 | 9.68 | 8.01 | 8.35 | 8.12 | 7.54 |
| Goethite | FeOOH | 6.66 | 5.78 | 6.75 | 5.78 | 6.58 | 6.07 | 6.81 | 6.56 | 7.52 | 6.76 | 6.92 | 6.72 | 6.58 |
| Jarosite(ss) | (K_0.77_Na_0.03_H_0.2_)Fe_3_(SO_4_)_2_(OH)_6_ | -2.03 | -4.70 | -2.08 | -4.91 | -3.19 | -4.48 | -1.83 | -3.67 | -1.10 | -2.15 | -2.86 | -2.02 | -3.35 |
| Jarosite-K | KFe_3_(SO_4_)_2_(OH)_6_ | -3.06 | -5.70 | -3.10 | -5.77 | -4.33 | -5.65 | -2.96 | -4.80 | -2.10 | -3.03 | -3.70 | -3.13 | -4.08 |
| Jarosite-Na | NaFe_3_(SO_4_)_2_(OH)_6_ | -5.56 | -8.15 | -5.59 | -8.18 | -7.77 | -9.06 | -6.47 | -8.50 | -5.33 | -5.99 | -6.59 | -6.73 | -6.73 |
| Maghemite | Fe_2_O_3_ | 5.85 | 4.08 | 6.04 | 3.93 | 5.77 | 4.78 | 6.26 | 5.77 | 7.59 | 5.92 | 6.27 | 6.04 | 5.45 |
| Melanterite | FeSO_4_.7H_2_O | -5.16 | -5.22 | -5.03 | -5.26 | -5.37 | -5.95 | -5.51 | -5.73 | -6.36 | -5.46 | -5.38 | -5.27 | -5.46 |
| Siderite | FeCO_3_ | 0.38 | 0.40 | 0.68 | 0.33 | 0.37 | -0.27 | -0.08 | 0.10 | -0.41 | 0.04 | 0.67 | 0.06 | 0.49 |
| Siderite(d)(3) | FeCO_3_ | 0.02 | 0.04 | 0.32 | -0.05 | 0.02 | -0.62 | -0.43 | -0.25 | -0.76 | -0.33 | 0.30 | -0.30 | 0.11 |
| **Manganese** |  |  |  |  |  |  |  |  |  |  |  |  |  |  |
| Manganite | MnOOH | -6.61 | -7.48 | -6.46 | -7.54 | -6.57 | -6.88 | -6.01 | -6.52 | -5.06 | -6.35 | -6.74 | -6.30 | -6.92 |
| Pyrochroite | Mn(OH)_2_ | -7.05 | -7.08 | -6.66 | -7.05 | -6.83 | -7.34 | -6.92 | -6.85 | -6.67 | -6.98 | -6.72 | -6.95 | -6.90 |
| Rhodochrosite | MnCO_3_ | -0.06 | -0.02 | 0.29 | -0.03 | -0.01 | -0.48 | -0.15 | -0.25 | -0.17 | -0.13 | -0.08 | -0.19 | 0.01 |
| **Others** |  |  |  |  |  |  |  |  |  |  |  |  |  |  |
| Anhydrite | CaSO_4_ | -1.37 | -1.38 | -1.36 | -1.41 | -1.93 | -2.14 | -1.72 | -2.18 | -2.01 | -1.85 | -2.03 | -1.60 | -1.64 |
| Aragonite | CaCO_3_ | -0.34 | -0.27 | -0.16 | -0.36 | -0.68 | -0.94 | -0.77 | -0.83 | -0.56 | -0.88 | -0.51 | -0.77 | -0.25 |
| Calcite | CaCO_3_ | -0.19 | -0.11 | -0.01 | -0.21 | -0.53 | -0.79 | -0.62 | -0.68 | -0.41 | -0.73 | -0.36 | -0.61 | -0.10 |
| Dolomite | CaMg(CO_3_)_2_ | -0.16 | -0.44 | -0.23 | -0.59 | -1.27 | -1.80 | -1.41 | -1.60 | -0.92 | -1.52 | -0.79 | -1.40 | -0.39 |
| Gypsum | CaSO_4_.2H_2_O | -1.12 | -1.12 | -1.10 | -1.16 | -1.67 | -1.89 | -1.46 | -1.92 | -1.76 | -1.60 | -1.78 | -1.35 | -1.39 |
| Ionic balance error |  | 0.94 | -0.63 | -2.38 | -0.14 | -0.39 | -2.65 | -0.79 | 0.13 | -0.91 | -0.79 | 0.65 | -2.46 | 0.54 |
| Ionic strength (mol/kgw) |  | 0.025 | 0.026 | 0.026 | 0.024 | 0.012 | 0.009 | 0.013 | 0.009 | 0.011 | 0.012 | 0.012 | 0.013 | 0.020 |

**Table S2** Stable isotopic compositions of mine water in the study area: (a) in Autumn 2019

| No. | Sample ID | Sampling date | δ^2^H (‰, VSMOW) | δ^18^O (‰, VSMOW) | *d =* δ^2^H – 8×δ^18^O (‰, VSMOW) |
| --- | --- | --- | --- | --- | --- |
| 1 | QU1 | 26.11.2019 | -51.2 | -7.38 | 7.81 |
| 2 | QU2 | 26.11.2019 | -51.1 | -7.51 | 9.04 |
| 3 | QU3 | 26.11.2019 | -51.8 | -7.52 | 8.31 |
| 4 | ROT | 26.11.2019 | -51.8 | -7.65 | 9.36 |
| 5 | CAR | 04.12.2019 | -51.0 | -7.44 | 8.55 |
| 6 | MGG | 14.11.2019 | -50.7 | -7.53 | 9.50 |
| 7 | VEP | 22.10.2019 | -50.4 | -7.37 | 8.57 |
| 8 | TTS | 22.10.2019 | -49.5 | -7.34 | 9.23 |
| 9 | SCH | 15.11.2019 | -51.3 | -7.50 | 8.66 |
| 10 | PAU | 14.11.2019 | -50.3 | -7.27 | 7.86 |
| 11 | RUD | 22.10.2019 | -49.6 | -7.46 | 10.01 |
| 12 | DOH | 22.10.2019 | -49.5 | -7.25 | 8.47 |
| 13 | BRA | 24.11.2019 | -50.9 | -7.51 | 9.24 |
| 14 | ET | 24.11.2019 | -51.2 | -7.62 | 9.71 |
| 15 | GED | 24.11.2019 | -51.9 | -7.75 | 10.12 |
| 16 | STE | 22.10.2019 | -52.9 | -7.40 | 6.35 |
| 17 | LOU | 04.12.2019 | -50.9 | -7.47 | 8.89 |
| 18 | HER | 04.12.2019 | -50.3 | -7.65 | 10.83 |
| 19 | TIS | 13.10.2019 | -49.5 | -7.41 | 9.78 |
| 20 | KHR | 04.12.2019 | -50.9 | -7.69 | 10.67 |
| 21 | NSS | 24.11.2019 | -52.1 | -7.88 | 10.95 |
| 22 | GH | 24.11.2019 | -51.2 | -7.51 | 8.90 |
| 23 | VER | 15.11.2019 | -51.7 | -7.62 | 9.21 |
| 24 | JOH | 15.11.2019 | -51.3 | -7.58 | 9.27 |
| 25 | FRA | 15.11.2019 | -51.5 | -7.50 | 8.48 |
|  | Max |  | -49.5 | -7.25 | 10.95 |
|  | Min |  | -52.9 | -7.88 | 6.35 |
|  | Average |  | -51.0 | -7.51 | 9.11 |

**Table S2** and (b) in Summer 2020

| No. | Sample ID | Sampling date | δ^2^H (‰, VSMOW) | δ^18^O (‰, VSMOW) | *d =* δ^2^H – 8×δ^18^O (‰, VSMOW) |
| --- | --- | --- | --- | --- | --- |
| 1 | QU1 | 29.07.2020 | -51.9 | -7.46 | 7.74 |
| 2 | QU2 | 29.07.2020 | -52.4 | -7.59 | 8.31 |
| 3 | QU3 | 29.07.2020 | -52.0 | -7.58 | 8.68 |
| 4 | ROT | 29.07.2020 | -52.2 | -7.48 | 7.60 |
| 5 | MGG | 21.07.2020 | -51.1 | -7.43 | 8.39 |
| 6 | VEP | 15.07.2020 | -49.6 | -7.10 | 7.19 |
| 7 | TTS | 21.07.2020 | -49.8 | -7.47 | 9.92 |
| 8 | SCH | 30.07.2020 | -51.6 | -7.66 | 9.70 |
| 9 | PAU | 21.07.2020 | -50.5 | -7.51 | 9.59 |
| 10 | RUD | 21.07.2020 | -50.0 | -7.37 | 9.02 |
| 11 | DOH | 21.07.2020 | -49.5 | -7.29 | 8.75 |
| 12 | BRA | 22.07.2020 | -51.1 | -7.59 | 9.70 |
| 13 | ET | 22.07.2020 | -51.9 | -7.58 | 8.72 |
| 14 | GED | 22.07.2020 | -52.1 | -7.65 | 9.17 |
| 15 | STE | 21.07.2020 | -50.2 | -7.34 | 8.48 |
| 16 | TIS | 22.07.2020 | -50.8 | -7.59 | 9.93 |
| 17 | NSS | 22.07.2020 | -51.6 | -7.73 | 10.19 |
| 18 | GH | 22.07.2020 | -50.5 | -7.57 | 10.08 |
| 19 | VER | 15.07.2020 | -51.0 | -7.39 | 8.12 |
| 20 | JOH | 30.07.2020 | -51.2 | -7.53 | 9.04 |
| 21 | FRA | 30.07.2020 | -51.2 | -7.68 | 10.27 |
|  | Max |  | -49.5 | -7.10 | 10.27 |
|  | Min |  | -52.4 | -7.73 | 7.19 |
|  | Average |  | -51.0 | -7.50 | 8.98 |

**Table S2** (c) Stable isotopic compositions of deep mine water (from Wedewardt, 1995)

| Coal mine name | Sample ID | Date | Level  (m) | δ^2^H (‰, VSMOW) | δ^18^O (‰, VSMOW) |
| --- | --- | --- | --- | --- | --- |
| Friedlicher Nachbar | ZW2 | 06.11.1992 | -165 | -51.9 | -7.64 |
| Robert Müser | ZW5 | 09.11.1992 | -445 | -50.0 | -7.62 |
| Robert Müser | ZW6 | 09.11.1992 | -60 | -54.0 | -7.73 |
| Heinrich | ZW19 | 12.11.1992 | -338 | -52.6 | -7.67 |
| Heinrich | ZW20 | 12.11.1992 | -338 | -53.0 | -7.71 |
| Heinrich | ZW21 | 12.11.1992 | -458 | -51.6 | -7.71 |

**Table S3** Stable isotopic compositions of rainwater in Bochum (March 2020 to March 2021)

| No. | Sampling date | δ^2^H (‰, VSMOW) | δ^18^O (‰, VSMOW) | *d =* δ^2^H – 8×δ^18^O (‰, VSMOW) |
| --- | --- | --- | --- | --- |
| 1 | March 2020 | -42.1 | -7.20 | 15.49 |
| 2 | April 2020 | -37.8 | -5.72 | 7.94 |
| 3 | May 2020 | -36.9 | -5.56 | 7.53 |
| 4 | June 2020 | -37.5 | -5.70 | 8.08 |
| 5 | July 01.07–17.07.2020 | -34.0 | -5.09 | 6.73 |
| 6 | July/August 18.07–17.08.2020 | -36.2 | -5.23 | 5.70 |
| 7 | September 2020 | -62.3 | -9.14 | 10.81 |
| 8 | October 2020 | -50.9 | -7.69 | 10.62 |
| 9 | November 2020 | -39.5 | -6.22 | 10.29 |
| 10 | December 2020 | -86.3 | -12.13 | 10.73 |
| 11 | January 2021 | -72.5 | -10.05 | 7.90 |
| 12 | February 2021 | -68.1 | -9.62 | 8.91 |
| 13 | March 2021 | -61.1 | -9.28 | 13.09 |


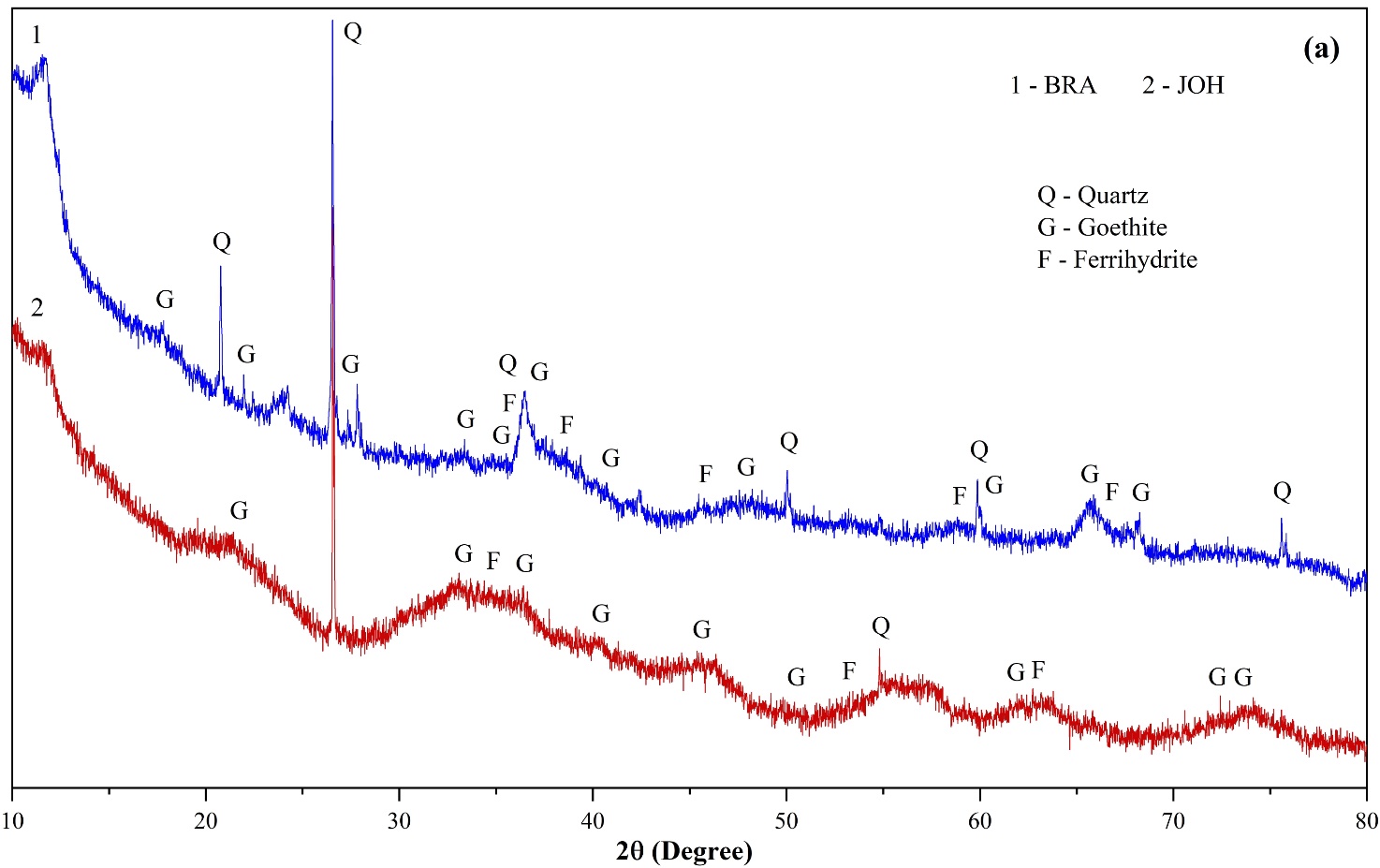


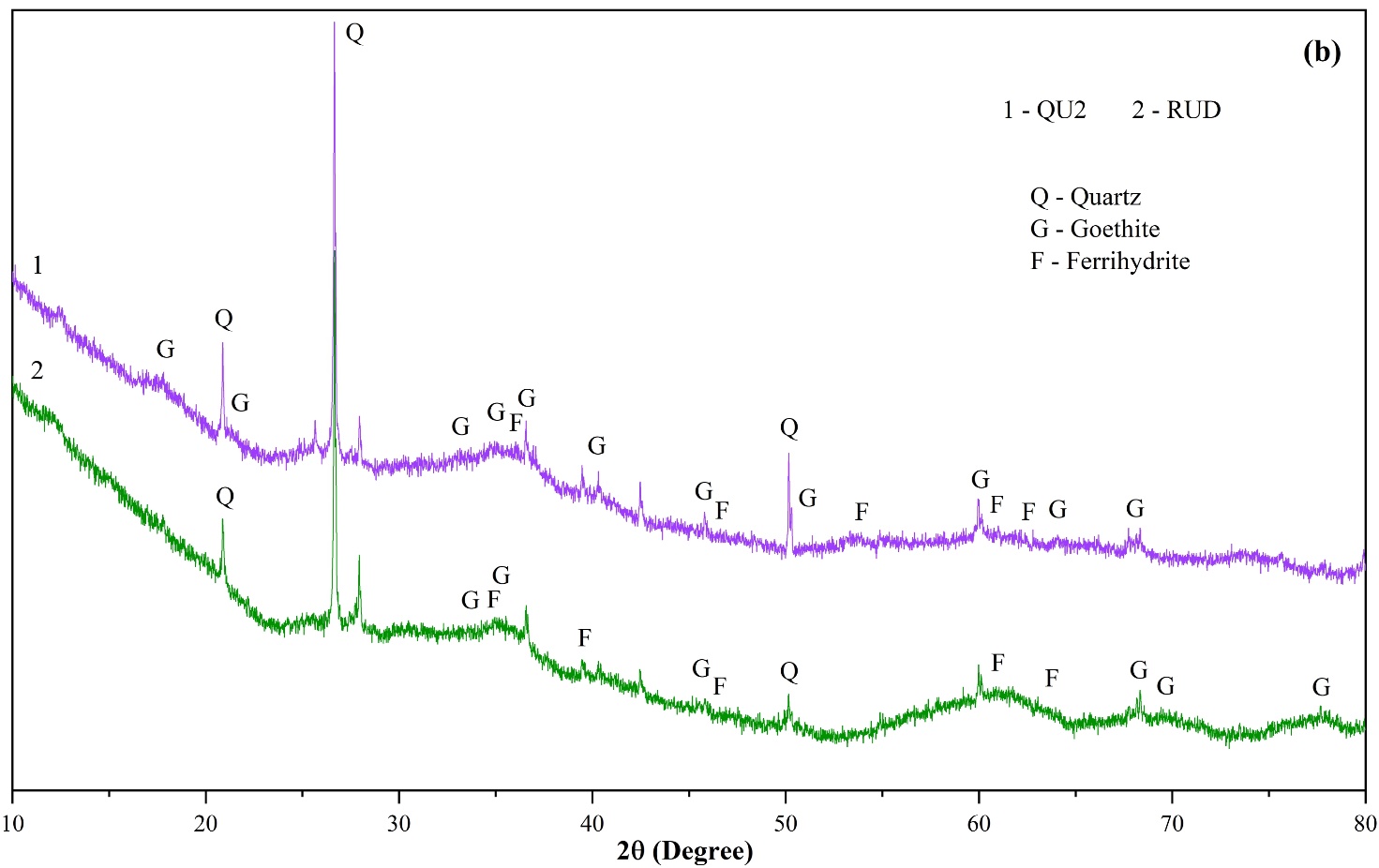


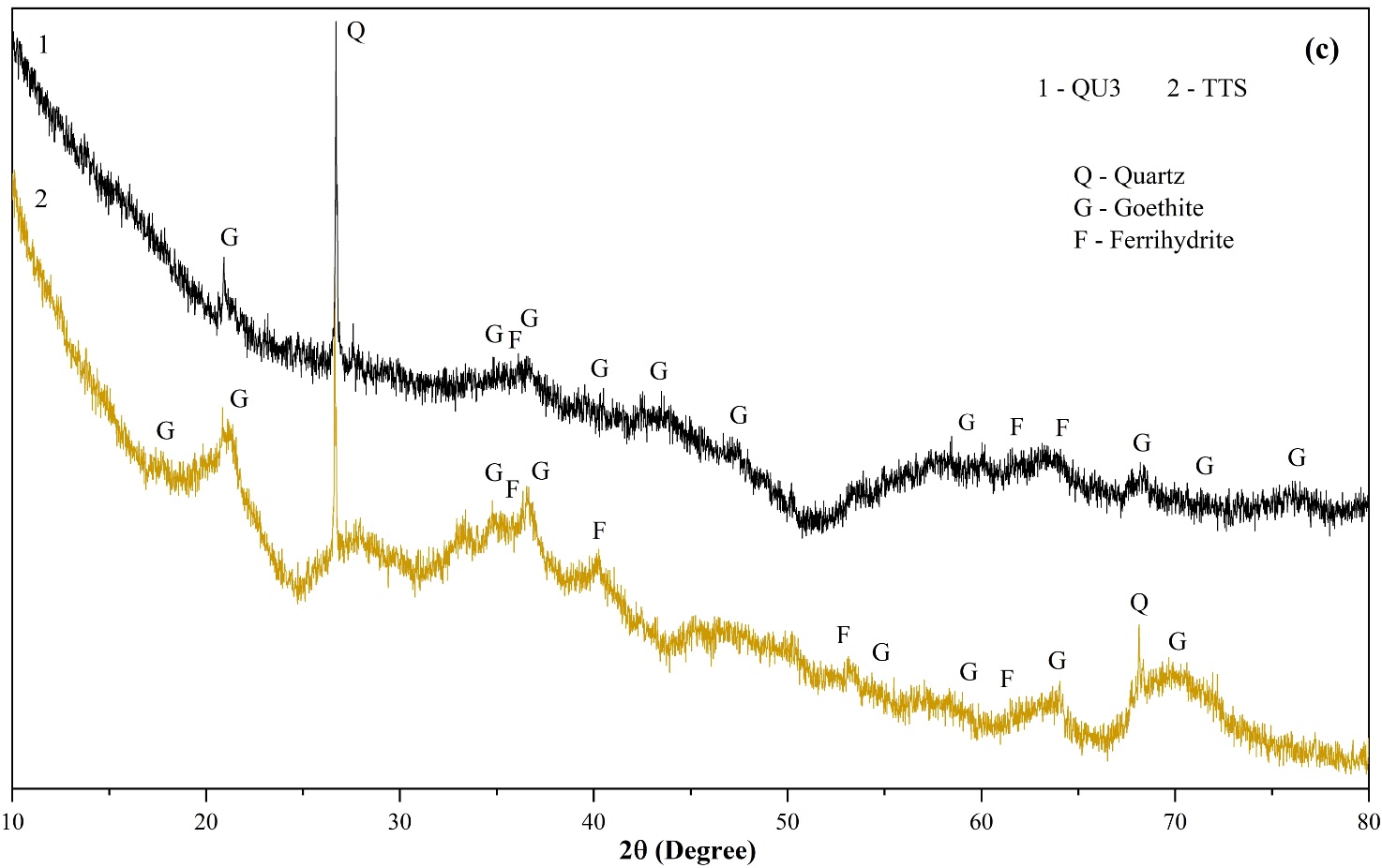


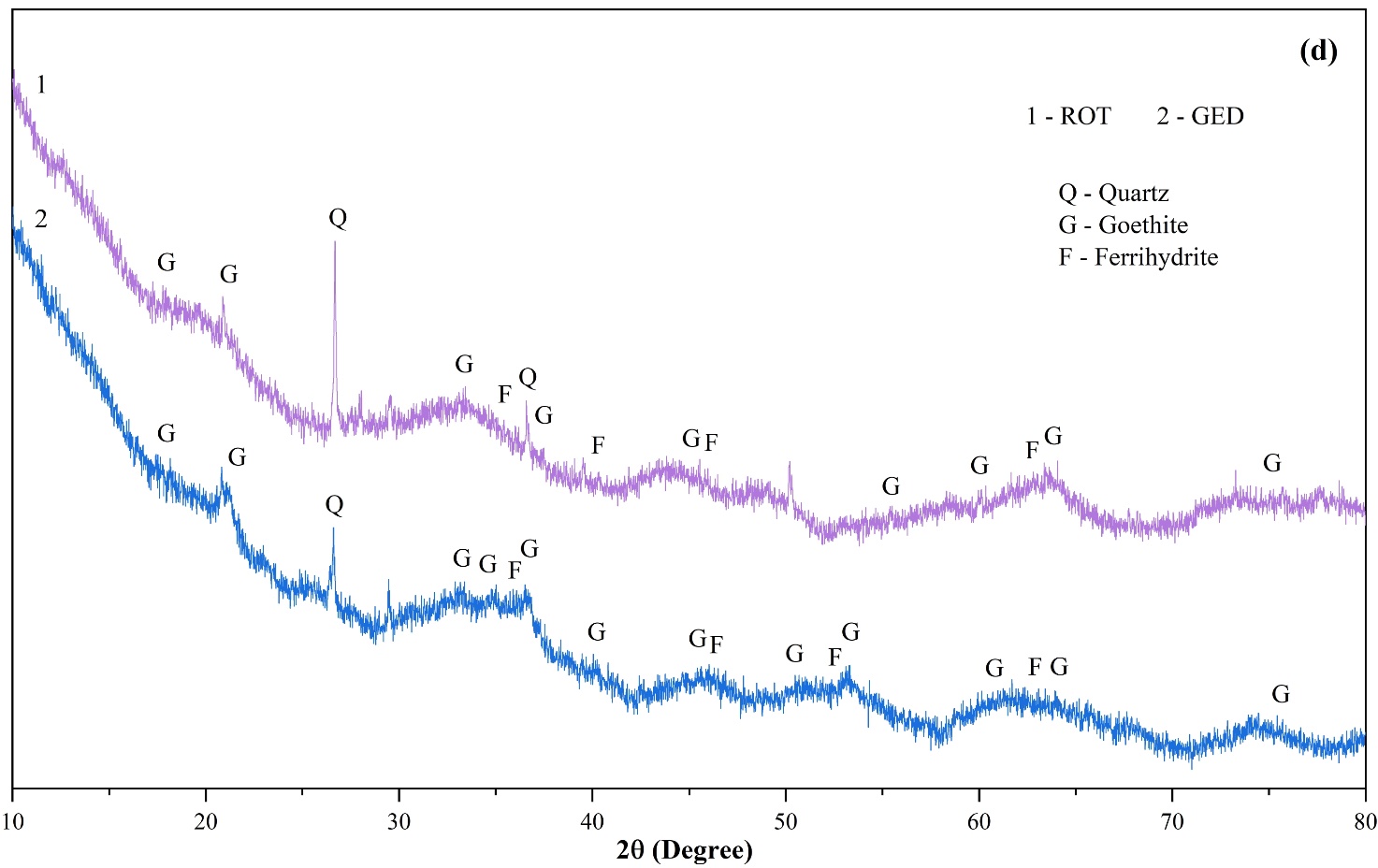


**Fig. S1** XRD analyses of ochreous precipitate samples (a) BRA and JOH, (b) QU2 and RUD, (c) QU3 and TTS, and (d) ROT and GED

**Fig. S2** Montmorillonite (Na,Ca)_0.3_(Al,Mg)_2_Si_4_O_10_(OH)_2_.n(H_2_O) coating Fe precipitates at the (a) BRA site and (b) ET site


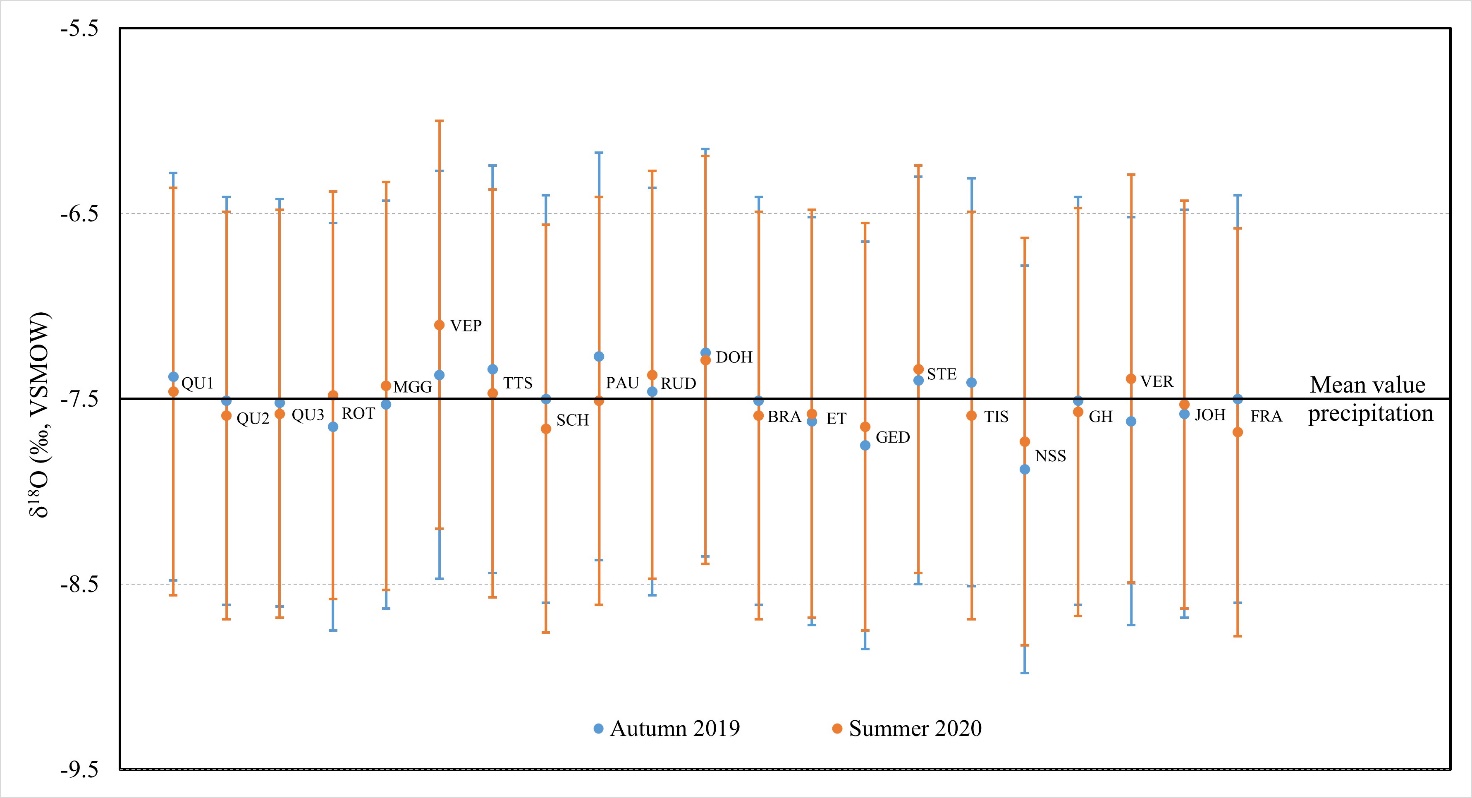


**(a)**


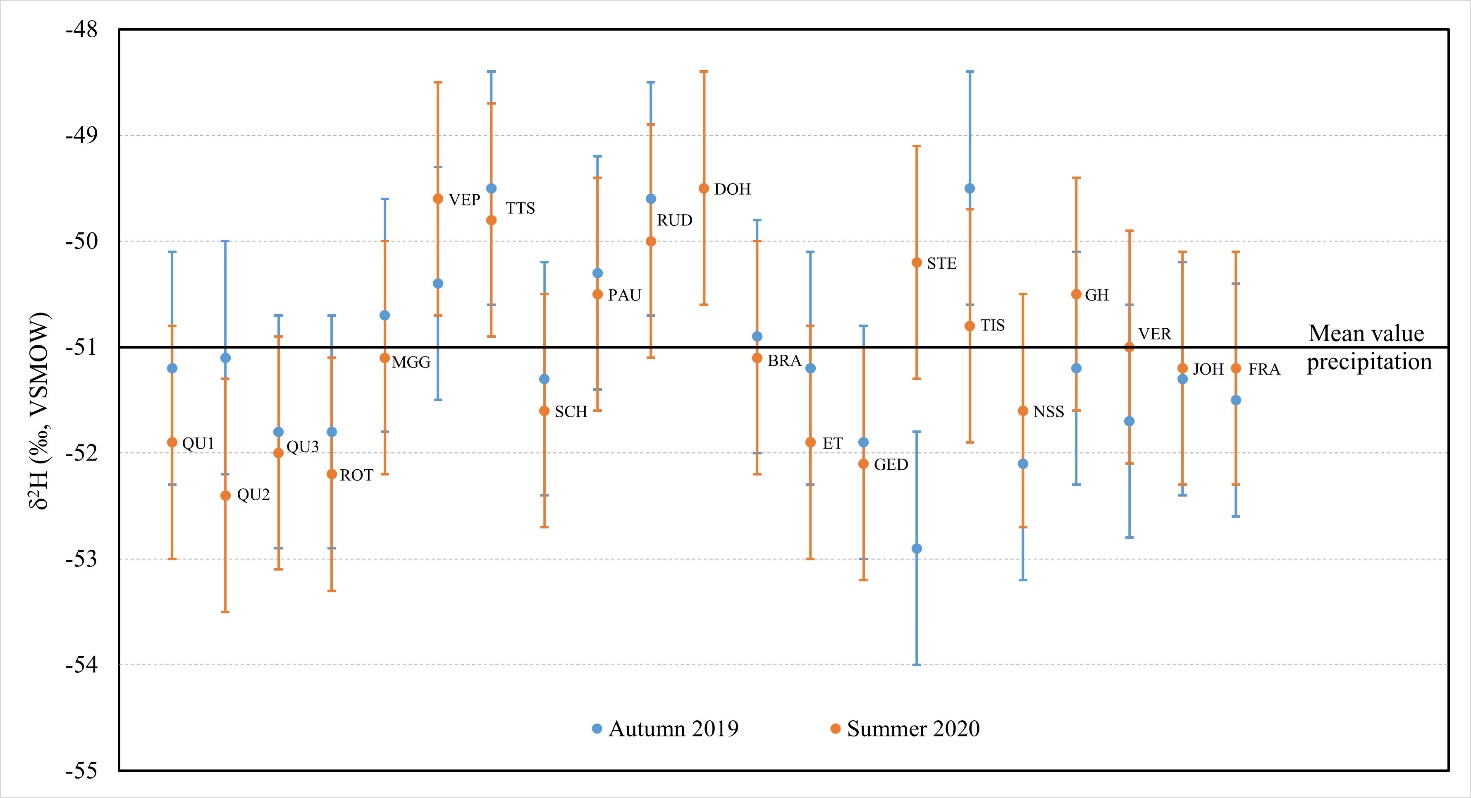


**(b)**

**Fig. S3** (a) δ^18^O and (b) δ^2^H values of mine waters collected at the same adit show similar values within the measurement error for autumn 2019 and summer 2020
